# miRNA and Degradome Sequencing Reveal miRNA and Their Target Genes That May Mediate Shoot Growth in Spur Type Mutant “Yanfu 6”

**DOI:** 10.3389/fpls.2017.00441

**Published:** 2017-03-30

**Authors:** Chunhui Song, Dong Zhang, Liwei Zheng, Jie Zhang, Baojuan Zhang, Wenwen Luo, Youmei Li, Guangfang Li, Juanjuan Ma, Mingyu Han

**Affiliations:** ^1^College of Horticulture, Yangling Subsidiary Center Project of National Apple Improvement Center, Collaborative Innovation Center of Shaanxi Fruit Industry Development, Northwest A&F UniversityYangling, China; ^2^Tongchuan Fruit Tree Experiment StationTongchuan, China

**Keywords:** apple, shoot, miRNAs, internode, SAM

## Abstract

The spur-type growth habit in apple trees is characterized by short internodes, increased number of fruiting spurs, and compact growth that promotes flowering and facilitates management practices, such as pruning. The molecular mechanisms responsible for regulating spur-type growth have not been elucidated. In the present study, miRNAs and the expression of their potential target genes were evaluated in shoot tips of “Nagafu 2” (CF) and spur-type bud mutation “Yanfu 6” (YF). A total of 700 mature miRNAs were identified, including 202 known apple miRNAs and 498 potential novel miRNA candidates. A comparison of miRNA expression in CF and YF revealed 135 differentially expressed genes, most of which were downregulated in YF. YF also had lower levels of GA, ZR, IAA, and ABA hormones, relative to CF. Exogenous applications of GA promoted YF shoot growth. Based on the obtained results, a regulatory network involving plant hormones, miRNA, and their potential target genes is proposed for the molecular mechanism regulating the growth of YF. miRNA164, miRNA166, miRNA171, and their potential targets, and associated plant hormones, appear to regulate shoot apical meristem (SAM) growth. miRNA159, miRNA167, miRNA396, and their potential targets, and associated plant hormones appear to regulate cell division and internode length. This study provides a foundation for further studies designed to elucidate the mechanism underlying spur-type apple architecture.

## Introduction

Apple is an important fruit crop in temperate regions of the world. The use of dwarfing rootstocks and high density planting of apple trees is common in commercial orchards as it increases yields per unit area and reduces the cost of management practices, such as pruning. The use of spur-type scion varieties is one of effective methods used to facilitate high-density planting, along with the use of dwarfing rootstocks.

“Fuji” is the major cultivar planted in China, accounting for more than 65% of apple plantings. Several spontaneous “Fuji” spur-type mutants have been identified and utilized in the breeding and planting of “Fuji” apples. The spur-type growth habit in apple is characterized by a reduced number of vegetative shoots and a corresponding increase in the number of fruiting spurs, short distances between nodes, compact and dense growth, and dark green and relatively thick leaves (Lapins and Lapins, 1969). Spur-type apple trees exhibit a high rate of bud break but have weak branching, characterized by rosette foliage and overall reduced levels of vegetative growth. The anatomical structure of spur-type shoots is characterized by vessel elements that are shorter and narrower than in standard-type shoots. The spur-type vessel elements have longer ends (tails), and thicker secondary cell walls (Yang et al., 2000).

Several earlier studies have reported that spur-type apple trees have lower levels of GA than standard-type trees, are less sensitive to applications of GA, and have reduced rates of conversion of GA19 to GA20 under high temperature conditions in summer (Steffens and Hedden, 1992; Song et al., 2012). The majority of shoot elongation is the result of cell expansion, with gibberellins playing a dominant role in this process. GAs promote internode elongation and overall shoot elongation by regulating cell proliferation, differentiation, and expansion. The “green revolution,” semi-dwarf varieties of cereal crops, are defective in GA biosynthesis and/or signaling. Auxin is recognized as the major hormone involved in shoot development (Gallavotti, 2013). Auxin also influences stem elongation and regulates the formation, activity, and fate of meristems. Cytokinins (CK) inhibit the differentiation of cells in the SAM and instead promotes cell division. CK promotes SAM enlargement partly through WUSCHEL, and defects in CK synthesis or perception reduces the size of the SAM size (Leibfried et al., 2006; Kyozuka, 2007). Except for GA, however, the levels of plant hormones in spur-type apples have not been reported or are unclear.

MicroRNAs (miRNAs) are a class of endogenous 20–24 nt non-coding RNAs. In plants, miRNAs are transcribed by RNA polymerase II, 5′ capped, spliced, and polyadenylated at the 3′ end to create pri-miRNAs. DICER-LIKE (DCL) protein processes the stem-loop structure in pri-miRNAs, creating pre-miRNA, and subsequently miRNA/miRNA^*^ duplexes. A single mature miRNAs is generated by the removal of the miRNA^*^. The mature miRNA is recruited into a RISC complex (RISC) which degrades mRNA targets or suppresses their translation. Plant miRNAs play a critical role in growth, organ development, phase changes, senescence, fruit ripening, stress response, disease resistance, stress tolerance, and mineral assimilation and translocation.

Some miRNAs in plants have been shown to regulate plant architecture. A point mutation in *OsSPL14* perturbs the cleavage of *OsSPL14* transcripts by OsmiR156, which affects tiller number and lodging resistance, as and enhances grain yield (Jiao et al., 2010). OsmiR397 down regulates a laccase-like protein *OsLAC* involved in brassinosteroid sensitivity, and thereby promotes panicle branching (Zhang et al., 2013). OsMIR444 participates in a miRNA/MADS/TCP/D14 (miMTD) regulation module, in which D14 functions in strigolactone (SL) signaling to control tillering, by suppressing *OsMADS57* expression (Guo et al., 2013). In pear trees, miR6390 is involved in the transition from endormancy to ecodormancy by targeting *PpDAM* transcripts, degrading them and stimulating the release of *PpFT2* (Niu et al., 2015). Overexpression of the *MbDRB1*, a gene involved in miRNA biogenesis in *Malus baccata*, increased the level of adventitious rooting, curly leaf, and columnar-like architecture (You et al., 2014).

The molecular mechanisms responsible for spur-type architecture is unclear. Few studies have been conducted on the role of miRNAs in the architecture of fruit trees. In the present study, miRNA and degradome sequencing was used to identify miRNA involved in shoot development in *Malus domestica* “Nagafu 2” and spur bud mutation “Yanfu 6.” Morphological indicies, plant hormone levels, miRNA, and target gene expression, and the application of various hormone treatments were also measured and/or used to compare and contrast shoot development in “Yanfu 6” vs. “Nagafu 2.” This comprehensive analysis was conducted in order to identify and determine the potential role of miRNA-mediated regulatory network in shoot growth and involvement of miRNA in the architecture of fruit trees and other woody, perennial plants.

## Materials and methods

### Plant materials

Experiments were conducted in 2013 and 2014 using standard-type “Nagafu 2” and spur-type mutant “Yanfu 6” apple (*Malus domestica*) trees that were grafted on Malling “M.26” rootstocks and planted in 2009. Trees were located in the experimental plots of the Yangling National Apple Improvement Center, Yangling, China (34.31° N, 108.04° E), trained and managed using standard horticultural practices. The meteorological conditions of the experiment site in 2013 and 2014 are described in Figure S1. Three biological replicates consisting of five trees of each genotype were used, for a total of 15 trees.

Apical portions (5–8 mm below and including the shoot meristem) of YF and CF shoots (without any leaves or petiole tissue), were collected at 45 days after bud break (DABB) in 2013 and used for deep miRNA sequencing. Similar apical portions of shoots were also collected at 65, 85, 105, 125, and 145 DABB in 2013 for miRNA and mRNA expression analysis. Approximately 30 shoot samples of each biological replicate of YF and CF were collected. Leaves near the shoot tips were also collected for plant hormone measurements. All samples were immediately frozen in liquid nitrogen and stored at −80°C for subsequent RNA and hormone extraction.

### Measurement of tree growth

Six individual shoots on each of six trees were marked in each genotype to assess shoot elongation on a weekly basis in 2013 and 2014. The number of internodes present on each shoot were also recorded. Internode lengths were estimated by dividing the overall shoot length by the number of internodes.

### Hormone measurements

Approximately 0.5 g of leaf samples were used to measure IAA, GA, ZR, and ABA. The plant hormones were extracted using the method described by Dobrev and Kamınek (2002) and Dobrev et al. (2005), quantifed by the High Pressure Liquid Chromatography (Waters, USA) and enzyme-linked immunosorbent assay.

### GA treatment

YF and CF trees were sprayed with 500 mg·L^−1^ GA_4+7_ at the end of April 2014. Shoot tips, as previously described, were subsequently sampled at 0, 2, 4, 6, and 8 days after spraying. Both shoot length and internode length were also recorded.

### Small RNA and degradome sequencing

Total RNA was isolated using the CTAB method (Chang et al., 1993). After ligating adaptors to the 5′ and 3′ RNA, the purified RNAs were reverse transcribed using primers with partial complementarity to the adaptors. The DNA pool amplified from the first-strand cDNA was subsequently sequenced using a Hiseq 2000 (Illumina, San Diego, CA) sequencer located at the Beijing Genomics Institute (BGI), Shenzhen.

The YF sample for miRNA sequencing was also subjected to degradome sequencing in order to better determine the miRNA/mRNA pairing relationship. For the construction of the degradome libraries, a 5′ RNA adapter with a *Mme* I recognition site was ligated to the 5′ terminus of purified polyA RNA. The 5′ ligated products were reverse transcribed using five PCR cycles, digested with *Mme* I, and ligated to a 3′ double DNA adapter. Lastly, the ligated products were amplified by PCR and gel-purified for deep sequencing.

The miRNA and degradome sequencing raw data were available at NCBI SRA (BioProject ID: PRJNA361540).

### Bioinformatic analysis of miRNA

The original sequence data from the sequencing machine were used for basic analysis. Filtering the low quality reads and contaminants sequence (adaptor, polyA, reads shorter than 18 nt, insert tag, reads without 3′ primer) and discarding the remaining reads with lengths smaller than 18 nt, the clean reads were used for the advanced analysis. The length distribution of the clean reads were summarized to analyze the compositions of small RNA sample. Unique tags and total tags existence among the sample were summarized to verify whether the uniformity of the two samples on the whole of sequencing was good.

The small RNA tags were mapped to the apple reference genome at GDR (http://www.rosaceae.org) (Jung et al., 2014) using SOAP software to analyze their distribution in the genome and their expression levels. All unique, clean reads, particularly the non-redundant reads, were subjected to further analysis. The clean tags were queried against the known apple miRNAs in the mirBASE 18.0 (http://www.mirbase.org) database in order to identify known microRNAs based on the following criteria: (1) the tags aligned to a miRNA precursor in the mirBASE database with no mismatch, and; (2) based on the first criteria, the tags aligned to mature miRNA in the mirBASE database with at least 16 nt overlap, allowing offsets. The unmatched unique reads in each sample were screened in Genbank in order to annotate non-coding RNA sequences (rRNA, scRNA, snoRNA, snRNA, and tRNA). Differentially expressed miRNAs were identified by comparing the sequencing reads between CF and YF and determining statistically significant differences (*P* < 0.05 and *P* < 0.01). miRNAs with more than one normalized read were analyzed by calculating their fold-change and *p* values. miRNAs with *p* < 0.05 and fold changes (log_2_) less than −1 or greater than 1 were considered to have significantly altered expression.

### Prediction of miRNA targets and functional annotation

The psRNATarget server (http://plantgrn.noble.org/psRNATarget/) with default parameters was used to predict putative targets of the identified apple miRNAs. All of the identified apple miRNA were used as a query against “*Malus domestica* (apple), predicted consensus gene set CDS sequences, version 1” database located on the psRNATarget server.

For the degradome sequencing raw data about 50 nt length obtained from HiSeq sequencing, go through steps likely adaptor, pollution, low quality trimmed to obtain “clean” sequence. After a series of annotation of the degradated sequence, the degradation mRNA obtained go through comparison with miRNA to find mRNA-miRNA pairing.

### GO enrichment and KEGG pathway analysis

GO enrichment and KEGG pathway analysis for all of the annotated miRNAs and their targets, using the apple reference genome background, were conducted in order to predict the function of the miRNA and their targets. Blast2GO was employed to store information from the GO and KEGG pathway databases. All of the miRNA targets were queried against the GO protein database (http://www.geneontology.org/) using BLASTX. A combined query was used in order to complete the GO annotation and pathway analysis against the GO and KEGG databases.

### Quantitative real-time PCR validation of microRNA and mRNA expression

Stem-loop RT-qPCR method was used to quantify miRNA expression (Chen et al., 2005). mRNAs of the target genes were also quantified. *5S rRNAs* were used as an internal reference of miRNA expression. *MdActin* and *MdEF* were used as internal references for mRNA expression. The primers utilized are listed in Tables S12, S13. miRNA and mRNA relative expression were calculated using the comparative ΔΔ*CT* method (Livak and Schmittgen, 2001).

### Statistical analysis

The data were analyzed using Data Processing System (DPS, version 7.05; Zhejiang University, Hangzhou, China) software. Significant differences were determined using a Student's *t*-test at (*p* ≤ 0.05).

## Results

### Growth characteristics of YF and CF

**Figure 1:**
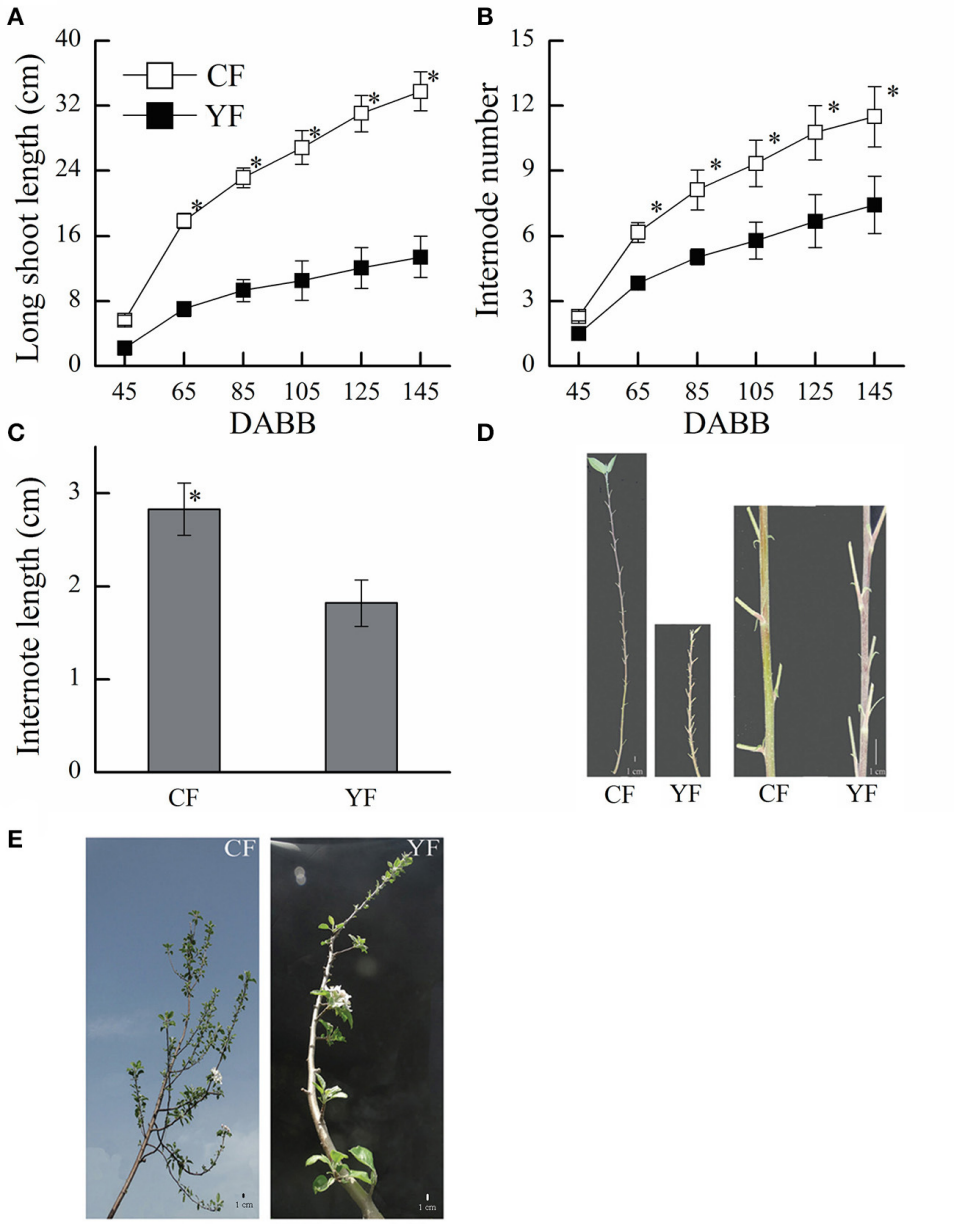
**Growth characteristics of CF and YF. (A)** Shoot length. **(B)** Internode number. **(C)** Internode length. **(D)** Shoot of Nagafu 2 (CF) and Yanfu 6 (YF). **(E)** Four-year-old branch of YF and CF. ^*^ indicates *P* < 0.05, Student's *t*-test; Error bars indicate SE; Scale bars in **(D,E)**, 1 cm.

The shoot length in YF was significantly shorter than in CF (Figures 1A,E). YF also had shorter internodes, that were approximately 66% shorter than CF (Figures 1C,D). Most YF shoots stopped growing between 80 and 105 DABB, which was earlier than CF shoots. After 105 DABB, any continued growth of YF shoots extended their length with a section of shorter internodes.

### Plant hormone analyses

**Figure 2:**
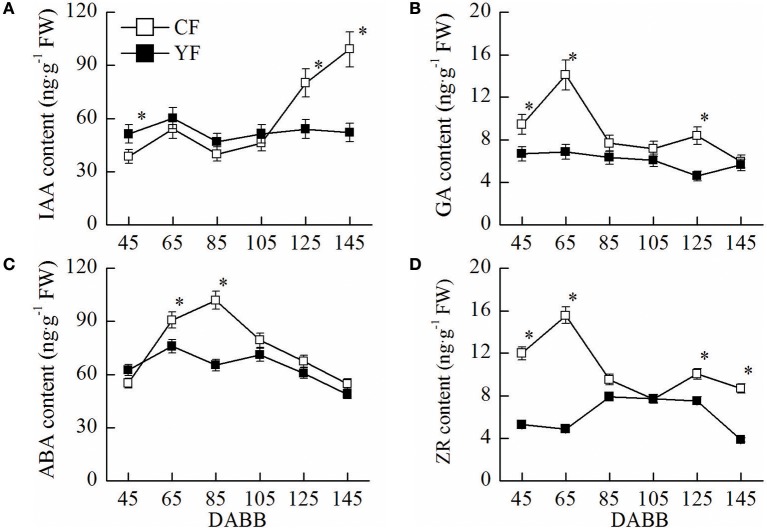
**Hormone content of CF and YF**. **(A)** IAA content. **(B)** GA content. **(C)** ABA content. **(D)** ZR content. Approximately 0.5 g leaves near shoot tips were used for plant hormone IAA, GA, ZR, and ABA measurements. ^*^Indicates *P* < 0.05, Student's *t*-test; Error bars indicate SE.

IAA in YF leaves near the shoot tips was higher than CF leaves at 45 DABB. In contrast, IAA levels in YF were significantly lower than CF at 125 and 145 DABB (Figure 2A). The GA content in YF leaves remained at a consistently low level, relative to CF, throughout the entire growth period (Figure 2B). The ABA content in YF was significantly lower than CF at 65 and 85 DABB (Figure 2C). The ZR content in YF was significantly lower than CF at 45, 65, 125, and 145 DABB (Figure 2D). The changes in the levels of the growth promoting hormones, IAA, GA, and ZR, were in accordance with the growth of shoot, namely, exhibiting a high content in the level during the rapid growth period, and a low level as the growth period waned (Figure 2). The stress hormone ABA exhibited the opposite trend, exhibiting a low level during the active growth period and a high level as growth slowed and terminated.

### Small RNA sequencing profiles in CF and YF

**Figure 3:**
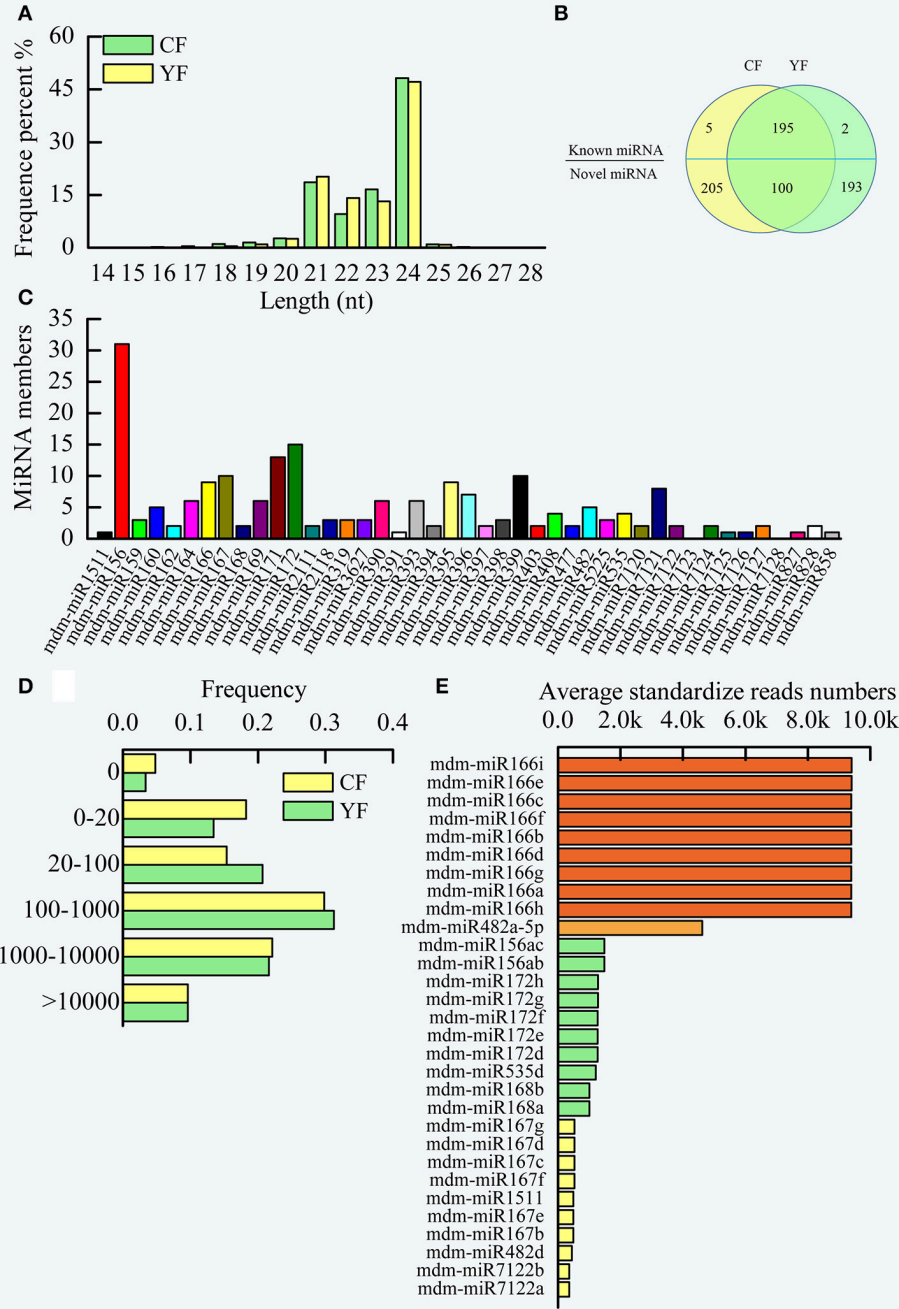
**Summary of miRNA in CF and YF shoot tips. (A)** The length distribution of small RNAs. **(B)** Venn diagrams of known and novel miRNAs in CF and YF shoot tips. **(C)** Known miRNA family members in CF and YF shoot tips. **(D)** miRNA reads counts frequency in CF and YF shoot tips. **(E)** Reads numbers of high expression miRNAs in shoot tips.

Two cDNA libraries of small RNAs from CF and YF were sequenced in order to identify miRNAs involved in apple shoot development. The sequencing produced a total of 13,264,874 raw reads from CF and 19,773,007 from YF (Table S1). After removal of the adaptor sequences, filtering out low-quality tags, and omitting contamination resulting from adaptor–adaptor ligations, 13,083,017 (98.95% of the total) CF and 19,580,358 (99.41%) YF clean reads remained, comprising 5,872,194 and 7,680,257 CF and YF unique sequences, respectively (Table S2). The unique sequences were mapped to the apple genome assembly using SOAP, resulting in 3,674,249 (62.57%) and 4,473,261 (58.24%) genome-matched reads for CF and YF, respectively. A total of 143,629 (2.44%) and 278,103 (2.54%) exon RNAs (sense and antisense), 267,025 (5.08%) and 321,431(4.18%) intron RNAs (sense and antisense), 130,796 (2.23%) and 204,833 (2.67%) rRNAs, 1,973,617 (33.61%) and 2,370,059 (30.86%) repeat regions, 4733 (0.08%) and 6054 (0.08%) snRNAs, 1,238 (0.02%) and 1,451 (0.02%) snoRNAs, 11,364 (0.19%), and 15,596 (0.20%) tRNAs, were removed from the CF and YF reads, respectively, This left 3,338,736 (56.86%) and 4,564,590 (59.43%) unannotated CF and YF RNAs, respectively, and a total of 1,056 (0.02%) and 1,149 (0.01%) miRNA reads for CF and YF, respectively. These were utilized as miRNA candidates in subsequent analyses. The read lengths in the two small RNA libraries were assessed (Figure 3A).The lengths ranged of the designated small RNAs ranged from 21 to 24 nt and accounted for more than 90% (approximately 92.9% and 94.6% of the CF and YF sequences, respectively) of all the clean reads in each library. The 24 nt small RNA was the most abundant small RNA length (about 50%). Similar results were reported in apple leaves, flowers, fruit, and roots (Xia et al., 2012), peach (*Prunus persica*) (Zhu et al., 2012), pear (*Pyrus bretschneideri*) (Wu et al., 2014), tomato (*Lycopersicon esculentum*) (Gao et al., 2015), and chickpea (Jain et al., 2014). The next most abundant small RNAs were 21 nt > 22 nt > 23 nt. There was a greater number of 23 nt and 24 nt small RNA in CF than in YF. The number of 21 and 22 nt small RNA were greater in YF than in CF.

The clean reads in each library were queried against the *Malus domestica* microRNA database in miRBase in order to identify the known miRNAs that were present in CF and YF apple shoot tips. Collectively, one million reads from the two libraries were perfectly matched to the *Malus domestica* microRNA database. A total of 202 unique sRNAs sequences were assigned to 41 microRNA families (Table S3, Figure 3B).

A comparison of family members revealed that the number of miRNA members varied among the different miRNA families (Figure 3C). The top five miRNA families, mdm-miR156, mdm-miR172, mdm-miR171, mdm-miR167, and mdm-miR399, had more than 10 members. For example, the mdm-miR156 family had 31 members. mdm-miR1511, mdm-miR391, mdm-miR7125, mdm-miR7126, mdm-miR827, and mdm-miR858 only had one member. miRNA156 is conserved in embryophytes (Zhang et al., 2006; Taylor et al., 2014) and represents the largest microRNA family in pear (Wu et al., 2014), which like apple, is a member of the Rosaceae. The differences in the number of members in the different microRNA families may be due to the different evolutionary rates in the microRNA families and to genome duplication events. Two known microRNAs families, mdm-miR7123, and mdm-miR7128, were not found to be present in the libraries (Figure 3C), therefore, these microRNAs may not be expressed in apple shoot tips. Statistical analysis of the read counts for the known miRNA families indicated significant differences in the level of expression among the different miRNAs (Table S3). Read counts of some miRNAs in a family were nearly the same, but some were obviously different. For example, all of the mdm-miR395 family members had the same number of counts. In contrast, mdm-miR172a, mdm-miR172o, and mdm-miR172g-h, members of the mdm-miR172 family (15 members), had a high read count, while mdm-miR172 a-c, mdm-miR172i-k, and mdm-miR172 m-o had a moderate read count, and mdm-miR172l had a low count. Perhaps some of the microRNA clustered in the same area of the genome, and thus had the same *cis*-regulation elements.

The read counts of about 30% of the known miRNAs were greater than 1,000 (Figure 3D). Another 30% of the known miRNA had read counts between 100 and 1,000. Due to high level of expression of miRNA in the apple shoot tip tissue, these miRNAs could be accurately identified. Approximately 20% of the known miRNA had read counts between 0 and 20.

Among the miRNA in YF and CF shoot tips, mdm-miRNA166 families exhibited the highest level of expression, with approximately 10,000 standardized reads (Figure 3E). This was followed by mdm-miR482a-5p, which had about 4000 standardized reads. The juvenile to adult phase transition related microRNAs, mdm-miR156 and mdm-miR172, and the flowering related, mdm-miR535, mdm-miR168, and mdm-miR167, also showed high expression levels in apple shoot tips.

After the identification of the known miRNAs, the unannotated tags were analyzed using MIREAP software in order to identify novel miRNAs based on the characteristics of the secondary hairpin structure of miRNA precursors. Using the hairpin structures of the precursors, 498 novel miRNAs were identified. The 23 nt length novel miRNAs represented an overwhelming amount 76.8% of the 306 total novel miRNAs identified (Table S4).

**Figure 4:**
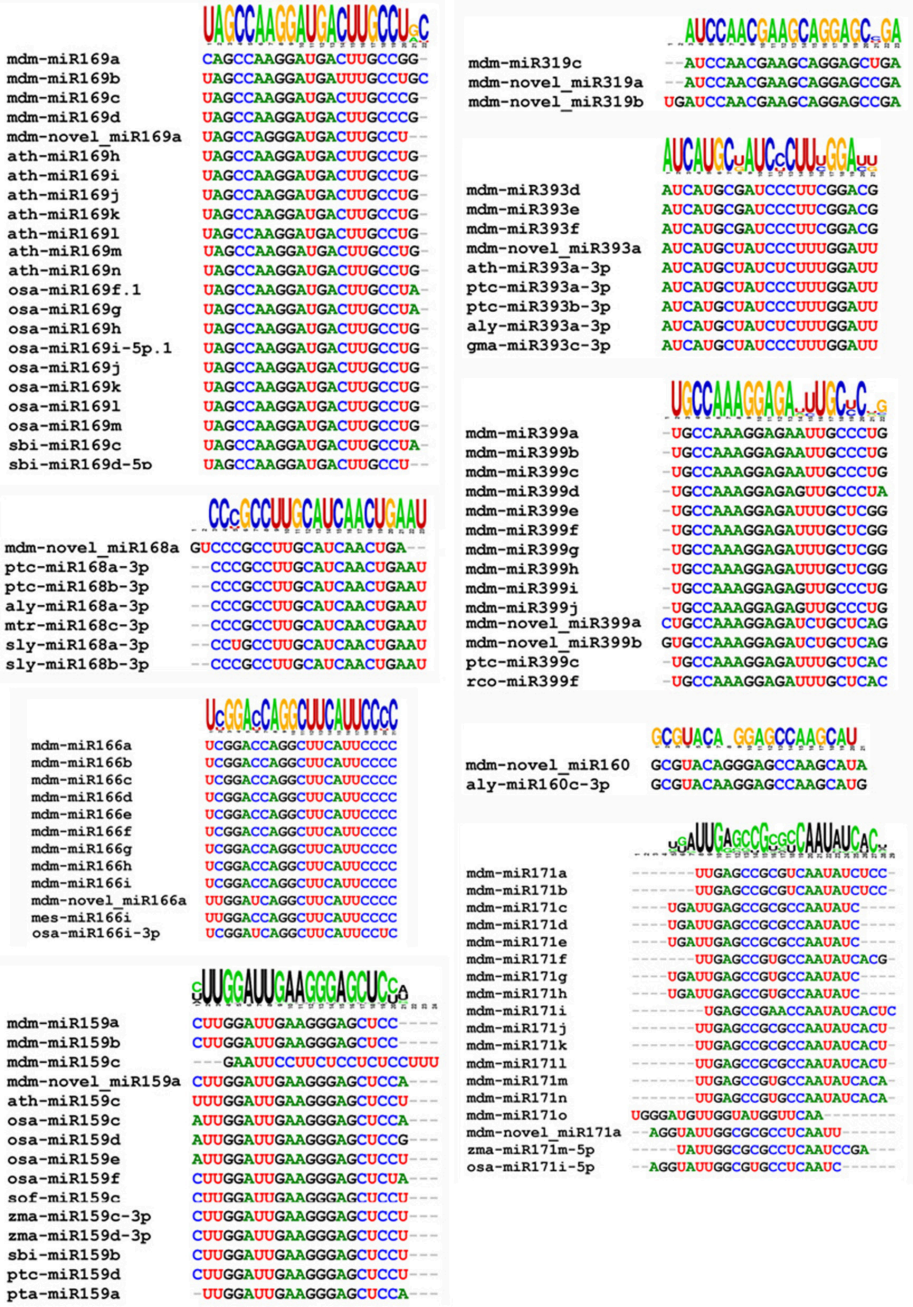
**Clustering of conserved and novel miRNAs into families**.

The novel miRNA was used a query to blast the miRBASE 18.0. Results indicated that 11 of the 498 novel miRNAs possessed a high significant similarity to known miRNAs from rice, wheat, and other plant species (Figure 4). Some varied only in nucleotide length, which may be due to differential cleavage sites in the pre-miRNA. These miRNAs represent new members of conserved miRNA families, and as a result, were designated as novel miRNA isoforms. The novel miRNAs would be species-specific *Malus domestica* miRNA.

Lower negative minimal folding free energy (MFE) value for RNA secondary structure, indicates greater stability (Bonnet et al., 2004). The average negative MFE was −51.03 kcal/mol (Table S4). This MFE value is similar to the data for *Arabidopsis thaliana* miRNA precursors (−57 kcal/mol) (Debat and Ducasse, 2014), chickpea (−57.58 kcal/mol) (Jain et al., 2014), maize (−61.15 kcal/mol) (Li et al., 2013), and pear (−51.48 kcal/mol) (Wu et al., 2014).

The GC content of miRNA has been shown to be related to biological function (Mishra et al., 2009). The GC content of 90% of the novel miRNA ranged between 30 and 70% (Table S4) with an average value of 42%. This average GC is similar to *Arabidopsis* (44%), pear (45%), and chickpea (44%), but less than grape (50%) and the GC content of the known miRNA of apple (50%).

The U residue is the most prevalent nucleotide at the first 5′ nucleotide site of miRNA, especially 21 nt miRNA (Chen, 2005). About 46% of the 5′ end nucleotides of the 21 nt novel miRNAs were U (Figure S2).

### Differentially expressed miRNAs

**Figure 5:**
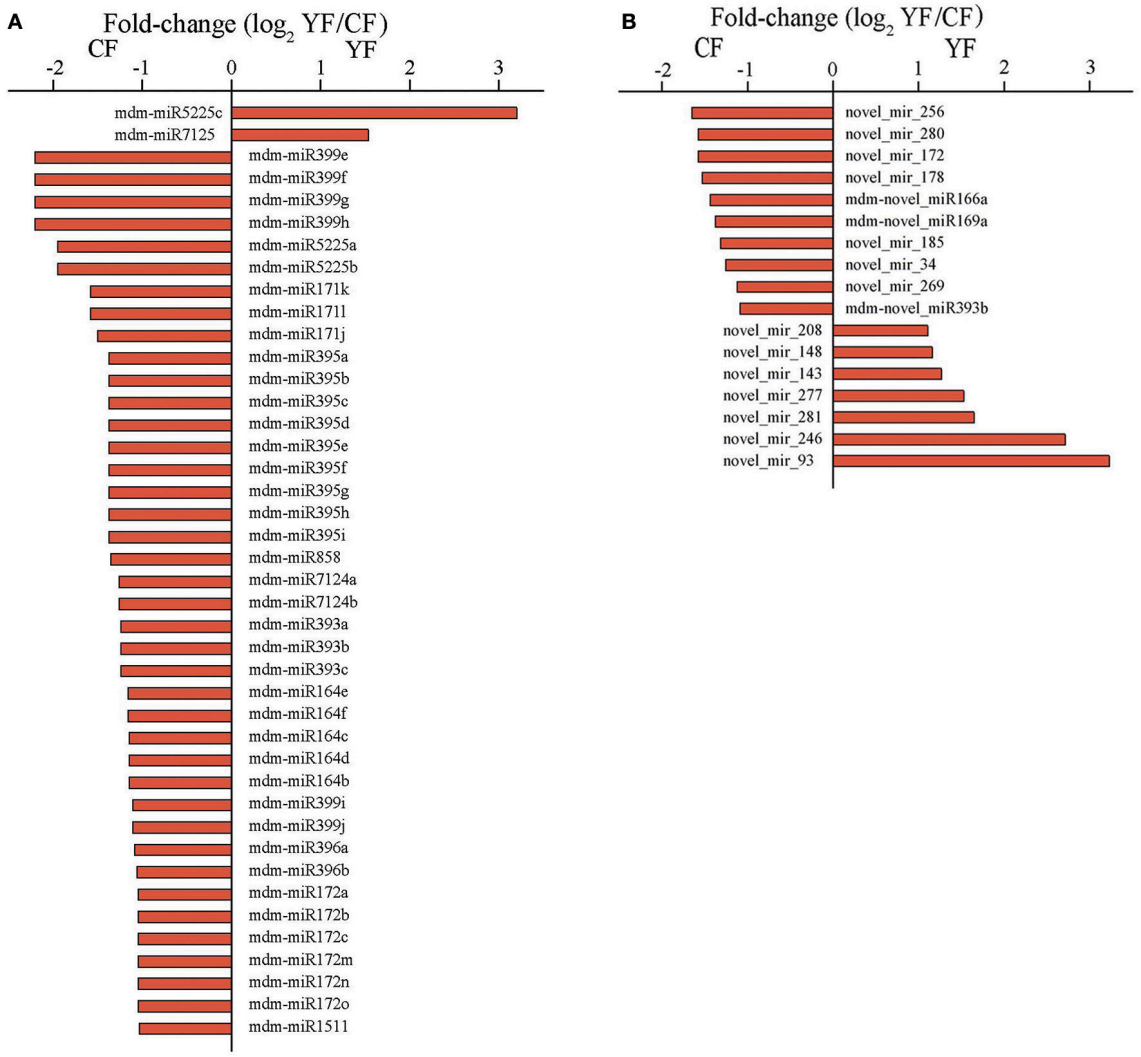
**Expression of miRNA in CF and YF shoot tips. (A)** Differential expression of known miRNAs in CF and YF shoot tips. **(B)** Differential expression of novel miRNAs in CF and YF shoot tips.

The abundances of miRNA reads in the CF and YF libraries were compared in order to identify miRNAs involved in shoot development and to determine their expression pattern in the two different genotypes with contrasting phenotypes. A total of 42 miRNAs, belonging to 12 known miRNA families, were identified whose expression differed significantly between the CF and YF shoot tips (Figure 5A). Among these, only mdm-miR5225c and mdm-miR7125 were upregulated in YF. In contrast, five members of mdm-miR164, three members of mdm-miR171, six members of mdm-miR172, three members of mdm-miR393, nine members of mdm-miR395, two members of mdm-miR396, six members of mdm-miR399, two members of mdm-miR5225, two members of mdm-miR7124, and mdm-miR858 were all downregulated in YF shoot tips.

Among of the 498 novel miRNAs, the expression of 93 differed significantly between CF and YF. A total of 34 upregulated and 59 were downregulated in YF (Figure 5B). A total of 49 novel miRNAs were expressed only in CF while 28 novel miRNAs were expressed only in YF (Table S4).

### Target prediction of miRNAs

**Figure 6:**
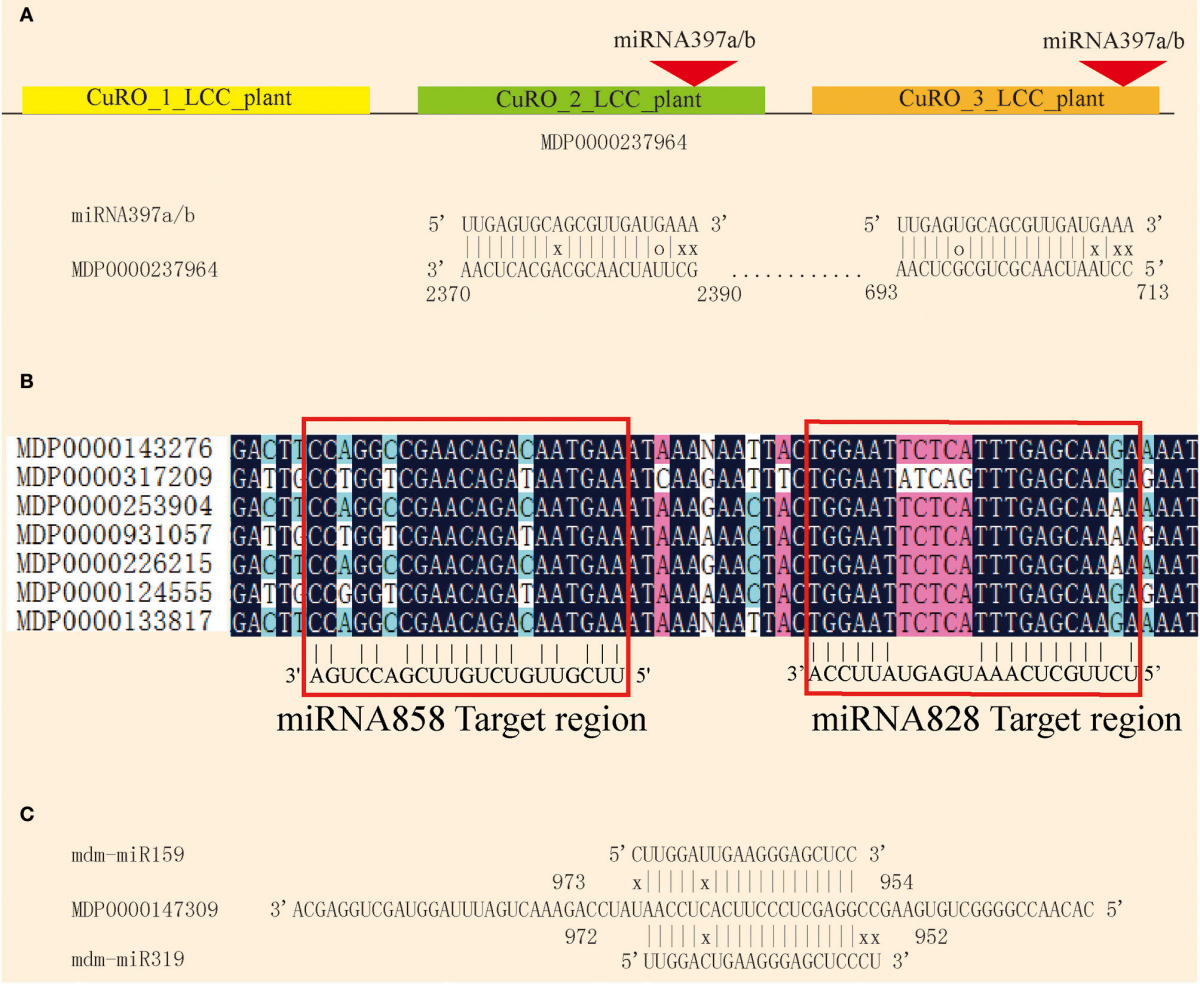
**Complementary targeting by miRNAs. (A)** miRNA397a/b targets the MDP0000237946 at two different sites. **(B)** miRNA828 and miRNA858 co-target the MYB conserved sequence. **(C)** miRNA159 and miRNA319 co-target MDP0000147309.

Plant miRNAs pair with their complementary target and inhibit translation or degrade the target mRNA. The complementary pairing of the miRNA/mRNA duplex provides a useful approach for target prediction. In order to better understand the role of apple miRNAs in shoot development, psRNATarget software was used to predict potential miRNA target genes. Using previously described criteria, 689 potential targets for 168 of the known miRNAs, and 3,663 targets for the 466 novel miRNAs were predicted (Tables S5, S6). The number of predicted targets for the various miRNAs varied from one to as many as 20, suggesting that these miRNAs may have diverse biological functions. On the other hand, a single gene may also be targeted by several miRNAs. MDP0000237964 encodes a LAC gene that is targeted by mdm-miR397 at two different sites, and two similar CuRO_LCC_plant domains (Figure 6A). miRNA828 and miRNA858 target the MYB family at the R3 domian (Xia et al., 2012; Figure 6B). MDP0000147309 is likely to be targeted by mdm-mi159 and mdm-mi319 (Figure 6C).

Degradome sequencing was conducted in order to better understand the role of miRNA-target regulation in shoot tips. Approximately 23 million total clean reads were generated from the degradome sequencing. About 49.5% of the unique reads mapped to apple mRNA (Table S7). The degradome sequencing results indicated that 246 genes are potentially regulated by 164 miRNAs, including both known and novel miRNAs. The main miRNAs and their target genes verified by degradome sequencing are listed in Tables 1, 2. Two-thirds of the degradome validated known miRNA target genes also identified in the target gene prediction software.

**Table 1:** **Identification potential target genes of known miRNA by the degradome sequencing**.

**miRNA**	**Target ID**	**Target function**	**Homologous At. gene**	**Abbreviation**
mdm-miR156	MDP0000146640	Squamosa promoter-binding protein-like family protein	AT1G69170	
mdm-miR156	MDP0000155354	Squamosa promoter binding protein-like 2	AT5G43270	SPL2
mdm-miR156	MDP0000249364	Acyl-CoA synthetase 5	AT1G62940	ACOS5
mdm-miR156	MDP0000297978	Squamosa promoter binding protein-like 9	AT2G42200	SPL9
mdm-miR156	MDP0000589558	Squamosa promoter-binding protein-like (SBP domain) transcription factor family protein	AT1G69170	
mdm-miR156	MDP0000778465	Squamosa promoter-binding protein-like (SBP domain) transcription factor family protein	AT1G69170	
mdm-miR159	MDP0000123466	Auxin response factor 2	AT5G62000	ARF2,
mdm-miR159	MDP0000147309	MYB domain protein 65	AT3G11440	MYB65
mdm-miR159	MDP0000237553	TEOSINTE BRANCHED 1, cycloidea and PCF transcription factor 2	AT4G18390	TCP2
mdm-miR159	MDP0000278072	Alpha/beta-Hydrolases superfamily protein	AT3G23600	
mdm-miR159	MDP0000287069	TEOSINTE BRANCHED 1, cycloidea and PCF transcription factor 2	AT4G18390	TCP2
mdm-miR159	MDP0000328318	TCP family transcription factor 4	AT3G15030	MEE35, TCP4
mdm-miR159	MDP0000442611	TCP family transcription factor 4	AT3G15030	MEE35, TCP4
mdm-miR159	MDP0000763497	TEOSINTE BRANCHED 1, cycloidea and PCF transcription factor 2	AT4G18390	TCP2
mdm-miR159	MDP0000916623	TCP family transcription factor 4	AT3G15030	MEE35, TCP4
mdm-miR159	MDP0000920127	TEOSINTE BRANCHED 1, cycloidea and PCF transcription factor 2	AT4G18390	TCP2
mdm-miR159	MDP0000927314	TEOSINTE BRANCHED 1, cycloidea and PCF transcription factor 2	AT4G18390	TCP2
mdm-miR160	MDP0000232116	Auxin response factor 17	AT1G77850	ARF17
mdm-miR160	MDP0000256621	Auxin response factor 17	AT1G77850	ARF17
mdm-miR160	MDP0000273491	Co-chaperone GrpE family protein	AT1G36390	
mdm-miR160	MDP0000750392	Auxin response factor 16	AT4G30080	ARF16
mdm-miR164	MDP0000121265	NAC domain containing protein 100	AT5G61430	NAC100,
mdm-miR164	MDP0000911724	NAC domain containing protein 100	AT5G61430	NAC100
mdm-miR166	MDP0000005879	Homeobox gene 8	AT4G32880	ATHB8,
mdm-miR166	MDP0000050082	Homeobox-leucine zipper family protein	AT2G34710	ATHB-1ATHB14, PHB
mdm-miR166	MDP0000126553	Homeobox gene 8	AT4G32880	ATHB8
mdm-miR166	MDP0000236500	Homeobox-leucine zipper family protein	AT5G60690	IFL, REV
mdm-miR166	MDP0000242861	Homeobox-leucine zipper family protein	AT5G60690	IFL, EV
mdm-miR166	MDP0000251484	Homeobox-leucine zipper family protein	AT1G52150	ATHB15, ICU4
mdm-miR166	MDP0000313059	Homeobox-leucine zipper family protein	AT1G52150	ATHB15, ICU4
mdm-miR166	MDP0000426630	Homeobox-leucine zipper family protein	AT5G60690	IFL, REV
mdm-miR166	MDP0000943529	Homeobox-leucine zipper family protein	AT2G34710	ATHB14, PHB
mdm-miR167	MDP0000321541	Phosphoglucomutase/phosphomannomutase family protein	AT1G70730	
mdm-miR167	MDP0000137461	Phosphoglucomutase/phosphomannomutase family protein	AT5G37020	ARF8
mdm-miR167	MDP0000153538	Auxin response factor 6	AT1G30330	ARF6
mdm-miR167	MDP0000232417	Auxin response factor 8	AT5G37020	ARF8
mdm-miR167	MDP0000268306	Auxin response factor 19	AT1G19220	ARF19
mdm-miR167	MDP0000319957	Auxin response factor 6	AT1G30330	ARF6
mdm-miR167	MDP0000550049	Auxin response factor 6	AT1G30330	ARF6
mdm-miR168	MDP0000069525	Stabilizer of iron transporter SufD / Polynucleotidyl transferase	AT1G48410	AGO1
mdm-miR168	MDP0000305971	Stabilizer of iron transporter SufD / Polynucleotidyl transferase	AT1G48410	AGO1
mdm-miR168	MDP0000352771			
mdm-miR169	MDP0000146933	Nuclear factor Y, subunit A9	AT3G20910	NF-YA9
mdm-miR169	MDP0000164531	Nuclear factor Y, subunit A7	AT1G30500	NF-YA7
mdm-miR169	MDP0000183865	Nuclear factor Y, subunit A7	AT1G30500	NF-YA7
mdm-miR169	MDP0000279028	Nuclear factor Y, subunit A7	AT1G30500	NF-YA7
mdm-miR169	MDP0000296077	Nuclear factor Y, subunit A1	AT5G12840	NF-YA1
mdm-miR171	MDP0000151144	GRAS family transcription factor	AT4G00150	HAM3
mdm-miR171	MDP0000274120	GRAS family transcription factor	AT4G00150	HAM3
mdm-miR171	MDP0000275704	GRAS family transcription factor	AT4G00150	HAM3
mdm-miR171	MDP0000784909	GRAS family transcription factor	AT2G45160	HAM1
mdm-miR172	MDP0000137561	Integrase-type DNA-binding superfamily protein	AT4G36920	AP2, FL1
mdm-miR172	MDP0000181606	Related to AP2.7	AT2G28550	RAP2.7, TOE1
mdm-miR172	MDP0000200319	Related to AP2.7	AT2G28550	RAP2.7, TOE1
mdm-miR172	MDP0000204900	Integrase-type DNA-binding superfamily protein	AT4G36920	AP2, FL1, FLO2
mdm-miR172	MDP0000281079	Related to AP2.7	AT2G28550	RAP2.7, TOE1
mdm-miR172	MDP0000285955	Related to AP2.7	AT2G28550	RAP2.7, TOE1
mdm-miR172	MDP0000296716	Related to AP2.7	AT2G28550	RAP2.7, TOE1
mdm-miR2118	MDP0000221561	NADP-malic enzyme 3	AT5G25880	ATNADP-ME3, NADP-ME3
mdm-miR319	MDP0000147309	MYB domain protein 65	AT3G11440	MYB65
mdm-miR319	MDP0000328318	TCP family transcription factor 4	AT3G15030	MEE35, TCP4
mdm-miR319	MDP0000442611	TCP family transcription factor 4	AT3G15030	MEE35, TCP4
mdm-miR319	MDP0000916623	TCP family transcription factor 4	AT3G15030	MEE35, TCP4
mdm-miR390	MDP0000353698			
mdm-miR393	MDP0000125975	F-box/RNI-like superfamily protein	AT3G62980	TIR1
mdm-miR393	MDP0000203334	Auxin signaling F-box 2	AT3G26810	AFB2
mdm-miR393	MDP0000268652	Auxin signaling F-box 3	AT1G12820	AFB3
mdm-miR393	MDP0000469943	Auxin signaling F-box 2	AT3G26810	AFB2
mdm-miR393	MDP0000498419	F-box/RNI-like superfamily protein	AT3G62980	TIR1
mdm-miR394	MDP0000576390	Geranylgeranyl pyrophosphate synthase 1	AT4G36810	GGPS1
mdm-miR394	MDP0000668869	Geranylgeranyl pyrophosphate synthase 1	AT4G36810	GGPS1
mdm-miR395	MDP0000121656	ATP sulfurylase 1	AT3G22890	APS1
mdm-miR395	MDP0000263161	ATP sulfurylase 1	AT3G22890	APS1
mdm-miR482	MDP0000130212	Chaperone DnaJ-domain superfamily protein	AT1G80920	J8
mdm-miR482	MDP0000160232	NB-ARC domain-containing disease resistance protein	AT3G14470	
mdm-miR482	MDP0000194077	Disease resistance protein (TIR-NBS-LRR class), putative	AT5G17680	
mdm-miR482	MDP0000210772	Disease resistance protein (TIR-NBS-LRR class) family	AT4G12010	
mdm-miR482	MDP0000222555	Chaperone DnaJ-domain superfamily protein	AT1G80920	J8
mdm-miR482	MDP0000287865	Disease resistance protein (TIR-NBS-LRR class) family	AT5G36930	
mdm-miR482	MDP0000304601	NB-ARC domain-containing disease resistance protein	AT3G14470	
mdm-miR482	MDP0000311052	Disease resistance protein (TIR-NBS-LRR class) family	AT5G36930	
mdm-miR482	MDP0000367358			
mdm-miR482	MDP0000810351	NB-ARC domain-containing disease resistance protein	AT3G14470	
mdm-miR482	MDP0000818967	Nuclear factor Y, subunit B3	AT4G14540	NF-YB3
mdm-miR482	MDP0000854581			
mdm-miR482	MDP0000196862	NSP-interacting kinase 3	AT1G60800	NIK3
mdm-miR482	MDP0000252094	NSP-interacting kinase 3	AT1G60800	NIK3
mdm-miR7122	MDP0000296050	Polyol/monosaccharide transporter 5	AT3G18830	ATPLT5
mdm-miR7122	MDP0000755167	Polyol/monosaccharide transporter 5	AT3G18830	ATPLT5
mdm-miR7125	MDP0000273257	Zinc transporter 1 precursor	AT3G12750	ZIP1
mdm-miR828	MDP0000124555	MYB domain protein 66	AT5G14750	MYB66
mdm-miR828	MDP0000133817	MYB domain protein 5	AT3G13540	MYB5
mdm-miR828	MDP0000167314	MYB domain protein 82	AT5G52600	MYB82
mdm-miR828	MDP0000226215	MYB domain protein 5	AT3G13540	MYB5
mdm-miR828	MDP0000253904	MYB domain protein 5	AT3G13540	MYB5
mdm-miR828	MDP0000261265	MYB domain protein 5	AT3G13540	MYB5
mdm-miR828	MDP0000264051	MYB domain protein 82	AT5G52600	MYB82
mdm-miR828	MDP0000578193	MYB domain protein 66	AT5G14750	MYB66, WER, WER1
mdm-miR828	MDP0000642761	MYB domain protein 66	AT5G14750	MYB66, WER, WER1
mdm-miR828	MDP0000650225	MYB domain protein 66	AT5G14750	MYB66, WER, WER1
mdm-miR828	MDP0000931057	High response to osmotic stress 10	AT1G35515	HOS10, MYB8
mdm-miR858	MDP0000031172	MYB domain protein 4	AT4G38620	MYB4
mdm-miR858	MDP0000124555	MYB domain protein 66	AT5G14750	MYB66, WER, WER1
mdm-miR858	MDP0000133817	MYB domain protein 5	AT3G13540	MYB5
mdm-miR858	MDP0000140609	MYB domain protein 12	AT2G47460	MYB12, PFG1
mdm-miR858	MDP0000143276	MYB domain protein 5	AT3G13540	MYB5
mdm-miR858	MDP0000184538	MYB domain protein 3	AT1G22640	MYB3
mdm-miR858	MDP0000210851	MYB domain protein 7	AT2G16720	MYB7
mdm-miR858	MDP0000226215	MYB domain protein 5	AT3G13540	ATMYB5, MYB5
mdm-miR858	MDP0000253904	MYB domain protein 5	AT3G13540	MYB5
mdm-miR858	MDP0000318013	Duplicated homeodomain-like superfamily protein	AT5G35550	MYB123, TT2
mdm-miR858	MDP0000437717	Duplicated homeodomain-like superfamily protein	AT5G35550	MYB123, TT2
mdm-miR858	MDP0000887107	MYB domain protein 12	AT2G47460	MYB12, PFG1
mdm-miR858	MDP0000931057	High response to osmotic stress 10	AT1G35515	HOS10, MYB8

**Table 2:** **Identification target of novel miRNA by the degradome sequencing**.

**miRNA**	**Target ID**	**Target function**	**Homologous At. gene**	**Abbreviation**
novel_mir_100	MDP0000360515	ATP synthase alpha/beta family protein	AT5G08680	
novel_mir_123	MDP0000253809	Annexin 2	AT5G65020	ANNAT2
novel_mir_124	MDP0000187925	UDP-glucose pyrophosphorylase 2	AT5G17310	ATUGP2, UGP2
novel_mir_144	MDP0000136541	MYB domain protein 105	AT1G69560	LOF2, MYB105
novel_mir_144	MDP0000146675	MYB domain protein 105	AT1G69560	LOF2, MYB105
novel_mir_144	MDP0000330904			
novel_mir_176	MDP0000368249			
mdm-novel_miR166a	MDP0000005879	Homeobox gene 8	AT4G32880	ATHB8
mdm-novel_miR166a	MDP0000050082	Homeobox-leucine zipper family protein / lipid-binding START domain-containing protein	AT2G34710	ATHB14, PHB
mdm-novel_miR166a	MDP0000126553	Homeobox gene 8	AT4G32880	ATHB8
mdm-novel_miR166a	MDP0000236500	Homeobox-leucine zipper family protein / lipid-binding START domain-containing protein	AT5G60690	IFL, REV
mdm-novel_miR166a	MDP0000242861	Homeobox-leucine zipper family protein / lipid-binding START domain-containing protein	AT5G60690	IFL, REV
mdm-novel_miR166a	MDP0000251484	Homeobox-leucine zipper family protein / lipid-binding START domain-containing protein	AT1G52150	ATHB15, ICU4
mdm-novel_miR166a	MDP0000313059	Homeobox-leucine zipper family protein / lipid-binding START domain-containing protein	AT1G52150	ATHB15, ICU4
mdm-novel_miR166a	MDP0000426630	Homeobox-leucine zipper family protein / lipid-binding START domain-containing protein	AT5G60690	IFL, REV
mdm-novel_miR166a	MDP0000943529	Homeobox-leucine zipper family protein / lipid-binding START domain-containing protein	AT2G34710	ATHB14, PHB
novel_mir_203	MDP0000161842	Ribosomal protein L4/L1 family	AT3G09630	
novel_mir_203	MDP0000680417	Ribosomal protein L4/L1 family	AT3G09630	
novel_mir_218	MDP0000665406	HAESA-like 1	AT1G28440	HSL1
mdm-novel_miR169a	MDP0000146933	Nuclear factor Y, subunit A9	AT3G20910	NF-YA9
mdm-novel_miR169a	MDP0000164531	Nuclear factor Y, subunit A7	AT1G30500	NF-YA7
mdm-novel_miR169a	MDP0000183865	Nuclear factor Y, subunit A7	AT1G30500	NF-YA7
mdm-novel_miR169a	MDP0000250518	Voltage dependent anion channel 1	AT3G01280	VDAC1
mdm-novel_miR169a	MDP0000279028	Nuclear factor Y, subunit A7	AT1G30500	NF-YA7
mdm-novel_miR169a	MDP0000296077	Nuclear factor Y, subunit A1	AT5G12840	NF-YA1
novel_mir_237	MDP0000095336	Serine protease inhibitor (SERPIN) family protein	AT1G47710	
novel_mir_237	MDP0000149531	Serine protease inhibitor (SERPIN) family protein	AT1G47710	
novel_mir_237	MDP0000210355	Serine protease inhibitor (SERPIN) family protein	AT1G47710	
novel_mir_237	MDP0000645731	Serine protease inhibitor (SERPIN) family protein	AT1G47710	
novel_mir_237	MDP0000751972	Serine protease inhibitor (SERPIN) family protein	AT1G47710	
novel_mir_249	MDP0000385086	Protein of unknown function (DUF1685)	AT4G33985	
novel_mir_249	MDP0000434028	Protein phosphatase 2A subunit A2	AT3G25800	PP2AA2
novel_mir_249	MDP0000509949	Peroxidase superfamily protein	AT1G05260	RCI3
novel_mir_249	MDP0000511597			
novel_mir_249	MDP0000540293	Protein phosphatase 2A subunit A2	AT3G25800	PP2AA2
novel_mir_249	MDP0000541775	Uncharacterised protein family (UPF0497)	AT3G23200	
novel_mir_249	MDP0000651056		AT5G05800	
novel_mir_249	MDP0000674456			
novel_mir_249	MDP0000690147	ARM repeat superfamily protein	AT4G16490	
novel_mir_249	MDP0000701629			
novel_mir_263	MDP0000165935	Nucleotide/sugar transporter family protein	AT5G17630	
novel_mir_266	MDP0000368249			
novel_mir_292	MDP0000530198	EXORDIUM like 3	AT5G51550	EXL3
novel_mir_292	MDP0000778016	EXORDIUM like 3	AT5G51550	EXL3
mdm-novel_miR159a	MDP0000147309	MYB domain protein 65	AT3G11440	MYB65
novel_mir_348	MDP0000297664	Mitochondrial substrate carrier family protein	AT5G19760	
novel_mir_384	MDP0000125404	Protein of unknown function, DUF538	AT1G02816	
novel_mir_384	MDP0000160246	Protein of unknown function, DUF538	AT1G02816	
novel_mir_384	MDP0000757026	Protein of unknown function, DUF538	AT1G02816	
novel_mir_388	MDP0000751972	Serine protease inhibitor (SERPIN) family protein	AT1G47710	
novel_mir_421	MDP0000751972	Serine protease inhibitor (SERPIN) family protein	AT1G47710	
novel_mir_447	MDP0000095336	Serine protease inhibitor (SERPIN) family protein	AT1G47710	
novel_mir_447	MDP0000149531	Serine protease inhibitor (SERPIN) family protein	AT1G47710	
novel_mir_447	MDP0000210355	Serine protease inhibitor (SERPIN) family protein	AT1G47710	
novel_mir_447	MDP0000645731	Serine protease inhibitor (SERPIN) family protein	AT1G47710	
novel_mir_447	MDP0000751972	Serine protease inhibitor (SERPIN) family protein	AT1G47710	
novel_mir_454	MDP0000265913	Farnesyl diphosphate synthase 1	AT5G47770	FPS1
novel_mir_454	MDP0000299402	Farnesyl diphosphate synthase 1	AT5G47770	FPS1
novel_mir_493	MDP0000126528	F-box family protein	AT4G24210	SLY1
novel_mir_497	MDP0000204850	Pentatricopeptide repeat (PPR) superfamily protein	AT3G53700	MEE40
novel_mir_58	MDP0000240422	Ribosomal protein L10 family protein	AT2G40010	
novel_mir_58	MDP0000398869	Ribosomal protein L10 family protein	AT2G40010	
novel_mir_58	MDP0000749281	Ribosomal protein L10 family protein	AT2G40010	
novel_mir_79	MDP0000243304	NB-ARC domain-containing disease resistance protein	AT3G14470	
novel_mir_80	MDP0000589558	Squamosa promoter-binding protein-like (SBP domain) transcription factor family protein	AT1G69170	
novel_mir_86	MDP0000320808	ENTH/VHS/GAT family protein	AT1G06210	
novel_mir_93	MDP0000231391	Leucine-rich repeat protein kinase family protein	AT2G01820	
novel_mir_93	MDP0000268423	Leucine-rich repeat protein kinase family protein	AT2G01820	

### GO and KEGG analysis of the degradome-predicted target genes in shoot tips

BLASTX querying against the protein database, Gene Ontology (GO), and KEGG pathway analysis were used to annotate the target genes identified in the degradome analysis in order to determine the potential functions of the miRNA target genes. A total of 69 degradome-identified target genes of 21 known miRNA were associated with 238 GO terms (Table S8). The first three enrichment GOs were GO:0005634, GO:0003700, and GO:0006351. GO:0005634, involved with the nucleus in the cellular component had 49 associated target genes. GO:0003700 involved in transcription factor activity in the molecular function component had 45 associated target genes. GO:0006351, involved in transcription, DNA-dependent in the biological process component had 41 associated target genes.

KEGG pathway analysis indicated that the target genes were involved in 21 pathways, in which, Transcription factors, ko03000; Metabolic pathways, ko01100, and; Plant hormone signal transduction, ko04075 were significantly enriched (Table S9).

Twenty-nine of the degradome identified genes targeted by 15 of the novel miRNAs were assigned to 176 GO terms (Table S10). Like the known miRNA, the GO enrichment in novel miRNA were transcription regulation related GO terms: 13 genes in GO:0005634; 9 genes in GO:0006355; 9 genes in GO:0003677, and; 7 genes in GO:0003700. KEGG pathway analysis indicated that the target genes were involved in 35 pathways, in which, Transcription factors, ko03000, and Metabolic pathways: ko01100 were significantly enriched. (Table S11).

YF exhibited a shorter duration of shoot elongation (Figure 1B). The duration of shoot growth is regulated by the shoot apical meristem (SAM). Based on the GO annotation of the target genes and with the KEGG pathway analysis, the potential targets of miR164, miR166, miR171, miR172, and miR482 are involved in meristem development (Table 3). In particular, miR164 participates in the formation of meristem-to-organ boundaries in the SAM (Laufs et al., 2004). The potential targets of miR166, miR159, and miR156 are involved in meristem phase transition. The potential targets of miR167, miR160, and miR159 play a role in auxin response and the auxin signaling pathway may also participate in SAM development.

**Table 3:** **SAM development relative miRNAs**.

**Development progress**	**miRNA**	**GO term**	**GO NO**.	**Target gene**
Primary shoot apical meristem specification	miR166, miR164	Primary shoot apical meristem specification	GO:0010072	MDP0000943529, MDP0000126553, MDP0000911724
Meristem development	miR167	Meristem development	GO:0048507	MDP0000232417, MDP0000550049, MDP0000319957
	miR167, miR166	Meristem initiation	GO:0010014	MDP0000232417, MDP0000319957, MDP0000313059, MDP0000005879, MDP0000943529, MDP0000126553, MDP0000251484
	miR172, miR167, miR166	Meristem maintenance	GO:0010073	MDP0000200319, MDP0000296716, MDP0000281079, MDP0000204900, MDP0000181606, MDP0000137561, MDP0000232417, MDP0000319957, MDP0000313059, MDP0000005879, MDP0000943529, MDP0000251484
	miR482, miR166	Regulation of meristem growth	GO:0010075	MDP0000196862, MDP0000313059, MDP0000005879, MDP0000943529, MDP0000251484
	miR166	Regulation of cell differentiation	GO:0045595	MDP0000126553
Phase transition of meristem	miR166, miR159, miR156	Vegetative to reproductive phase transition of meristem	GO:0010228	MDP0000126553, MDP0000442611, MDP0000916623, MDP0000297978
Plant hormone related	miR393	Auxin binding	GO:0010011	MDP0000268652, MDP0000203334, MDP0000125975
	miR393, miR167, miR160, miR159	Auxin mediated signaling pathway	GO:0009734	MDP0000268652, MDP0000498419, MDP0000203334, MDP0000125975, MDP0000232417, MDP0000268306, MDP0000550049, MDP0000319957, MDP0000232116, MDP0000123466
	miR858, miR159	Response to abscisic acid stimulus	GO:0009737	MDP0000318013, MDP0000123466
	miR858, miR393, miR167, miR166	Response to auxin stimulus	GO:0009733	MDP0000210851, MDP0000125975, MDP0000232417, MDP0000268306, MDP0000550049, MDP0000319957, MDP0000126553
	miR858, miR167	Response to ethylene stimulus	GO:0009723	MDP0000210851, MDP0000268306
	miR858, miR393	Ethylene mediated signaling pathway	GO:0009873	MDP0000318013, MDP0000125975
	miR167	Response to brassinosteroid stimulus	GO:0009741	MDP0000268306
Flower ogran development	miR172, miR167, miR159	Ovule development	GO:0048481	MDP0000200319, MDP0000296716, MDP0000281079, MDP0000204900, MDP0000181606, MDP0000137561, MDP0000232417, MDP0000550049, MDP0000319957, MDP0000442611, MDP0000123466, MDP0000916623
	miR166	Polarity specification of adaxial/abaxial axis	GO:0009944	MDP0000313059, MDP0000005879, MDP0000943529, MDP0000126553, MDP0000251484
	miR169, miR160	Pollen development	GO:0009555	MDP0000296077, MDP0000232116
	miR156	Pollen exine formation	GO:0010584	MDP0000249364
	miR393	Pollen maturation	GO:0010152	MDP0000268652, MDP0000203334, MDP0000125975
	miR159	Pollen sperm cell differentiation	GO:0048235	MDP0000147309
	miR2118	Pollen tube growth	GO:0009860	MDP0000221561
	miR167	Regulation of flower development	GO:0009909	MDP0000232417, MDP0000550049, MDP0000319957
	miR393	Stamen development	GO:0048443	MDP0000268652, MDP0000203334, MDP0000125975
	miR172, miR167	Flower development	GO:0009908	MDP0000200319, MDP0000296716, MDP0000281079, MDP0000204900, MDP0000181606, MDP0000137561
	miR167, miR166	Flower morphogenesis	GO:0048439	MDP0000232417, MDP0000550049, MDP0000319957, MDP0000313059, MDP0000005879, MDP0000251484
	miR172, miR166	Tissue development	GO:0009888	MDP0000200319, MDP0000296716, MDP0000281079, MDP0000204900
Embryonic development	miR396	Embryonic axis specification	GO:0000578	MDP0000168690
	miR396, miR169	Embryonic development	GO:0009790	MDP0000168690, MDP0000296077
	miR166, miR159	Embryonic development ending in seed dormancy	GO:0009793	MDP0000126553, MDP0000442611, MDP0000916623
	miR166	Embryonic pattern specification	GO:0009880	MDP0000943529, MDP0000126553
Formation of organ boundary	miR164	Formation of organ boundary	GO:0010160	MDP0000911724
	miR166	Polarity specification of adaxial/abaxial axis	GO:0009944	MDP0000313059, MDP0000005879, MDP0000943529, MDP0000126553, MDP0000251484
	miR167, miR166	Determination of bilateral symmetry	GO:0009855	MDP0000232417, MDP0000550049, MDP0000319957, MDP0000313059, MDP0000005879, MDP0000943529
	miR166	Determination of dorsal identity	GO:0048263	MDP0000313059, MDP0000005879, MDP0000251484

Internode length is determined by cell division and cell elongation. Based on the GO annotation of the target genes and the KEGG pathway analysis, miRNAs related to cell division and cell differentiation were identified (Table 4). The potential targets of miR159, miR166, miR167, miR171, miR172, miR393, miR858, and miR828 are involved in cell growth. Gibberellin also plays an important role in regulating internode length. The potential targets of miR159 are involved in the gibberellic acid mediated signaling pathway and gibberellin biosynthetic process. While the potential targets of miRNA160 and miRNA167 regulate auxin response to control cell division. Thus, the potential targets of miR167, involved in response to brassinosteroid stimulus and the auxin-mediated signaling pathway, may play a role in internode development. Secondary metabolism affects primary growth by competing for common substrates. The potential targets of miR858, miR828, and miR156 participate in the fatty acid biosynthetic process. Cell walls also affect cell elongation and cell size. The potential targets of miR166 and miR167 are involved in cell wall biogenesis and cell wall organization. Mineral nutrition affects plant physiology and growth and the potential targets of miR169, miR166, miR7125, miR393, and miR395 are involved in mineral element response.

**Table 4:** **Internode growth related miRNAs**.

**Development progress**	**miRNA**	**GO term**	**GO NO**.	**Target gene**
Cell growth	miR167, miR166	Cell adhesion	GO:0007155	MDP0000268306, MDP0000313059, MDP0000005879, MDP0000943529, MDP0000126553, MDP0000251484
	miR393	Cell cycle	GO:0007049	MDP0000125975
	miR172, miR171, miR166, miR159	Cell differentiation	GO:0030154	MDP0000200319, MDP0000296716
	miR171, miR166	Cell division	GO:0051301	MDP0000275704, MDP0000151144, MDP0000784909, MDP0000126553
	miR858, miR828	Cell fate commitment	GO:0045165	MDP0000184538, MDP0000578193, MDP0000261265, MDP0000650225, MDP0000124555
	miR858, miR828	Cell fate specification	GO:0001708	MDP0000184538, MDP0000578193, MDP0000931057, MDP0000261265, MDP0000650225, MDP0000124555
	miR166	Cell growth	GO:0016049	MDP0000126553
	miR166	Cell wall biogenesis	GO:0042546	MDP0000126553
	miR166	Cell wall macromolecule metabolic process	GO:0044036	MDP0000126553
	miR167, miR166	Cell wall organization	GO:0071555	MDP0000268306, MDP0000313059, MDP0000005879, MDP0000943529, MDP0000126553, MDP0000251484
Xylem development	miR167, miR166	Xylem and phloem pattern formation	GO:0010051	MDP0000232417, MDP0000550049, MDP0000319957, MDP0000313059, MDP0000005879, MDP0000943529, MDP0000251484
	miR166	Xylem development	GO:0010089	MDP0000313059, MDP0000005879, MDP0000126553, MDP0000251484
Gibberellin	miR159	Gibberellic acid mediated signaling pathway	GO:0009740	MDP0000147309
	miR159	Gibberellin biosynthetic process	GO:0009686	MDP0000147309
Fatty acid biosynthetic process	miR858, miR828	Fatty acid biosynthetic process	GO:0006633	MDP0000887107, MDP0000437717, MDP0000226215, MDP0000253904
	miR156	Fatty-acyl-CoA biosynthetic process	GO:0046949	MDP0000249364
Flavonoid biosynthetic process	miR858, miR828	Flavonoid biosynthetic process	GO:0009813	MDP0000210851, MDP0000887107, MDP0000437717, MDP0000251486
Ethylene mediated signaling pathway	miR393	Ethylene mediated signaling pathway	GO:0009873	MDP0000125975
	miR858, miR393	Ethylene mediated signaling pathway	GO:0009873	MDP0000318013, MDP0000125975
Cell wall development	miR166	Cell wall biogenesis	GO:0042546	MDP0000126553
	miR166	Cell wall macromolecule metabolic process	GO:0044036	MDP0000126553
	miR167, miR166	Cell wall organization	GO:0071555	MDP0000268306, MDP0000313059, MDP0000005879, MDP0000943529, MDP0000251487
Mineral nutrition	miR7125	Cellular response to iron ion starvation	GO:0010106	MDP0000273257
	miR393	Cellular response to nitrate	GO:0071249	MDP0000268652, MDP0000203334
	miR166	Cellular response to nitrogen starvation	GO:0006995	MDP0000005879, MDP0000251484, MDP0000313059
	miR393, miR169	Cellular response to phosphate starvation	GO:0016036	MDP0000125975, MDP0000164531
	miR395	Sulfate accumulation and allocation		MDP0000263161, MDP0000121656
Auxin binding	miR393	Auxin binding	GO:0010011	MDP0000268652, MDP0000203334, MDP0000125975
	miR393, miR167, miR160, miR159	Auxin mediated signaling pathway	GO:0009734	MDP0000268652, MDP0000498419, MDP0000203334, MDP0000125975, MDP0000232417, MDP0000268306, MDP0000251490

### RT-qPCR of differentially expressed miRNAs and their targets involved in SAM development

**Figure 7:**
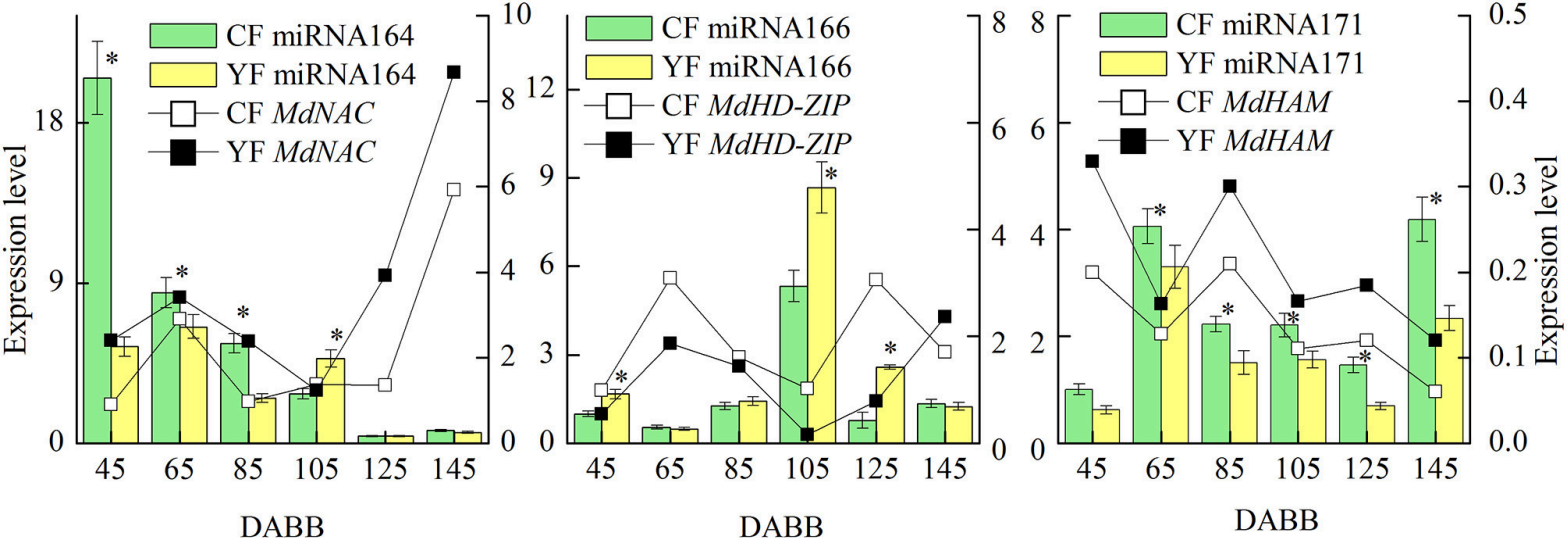
**Differentially expressed miRNAs and their targets involved in SAM development between CF and YF**. ^*^Indicates *P* < 0.05, Student's *t*-test; Error bars indicate SE.

The miRNAs and their targets putatively involved in SAM development that were differentially expressed in YF vs. CF are illustrated in Figure 7. The expression analysis of miRNAs and their associated target genes revealed inversed expression patterns. The expression of miRNA164 decreased from 45 to 145 DABB in both YF and CF while expression of the potential target gene, *MdNAC*, increased from 45 to 55 DABB, decreased from 85 to 105 DABB, and then increased rapidly after 125 DABB. The expression of miRNA164 in YF was significantly lower than in CF at 45, 65, and 85 DABB. The expression of miRNA166 in CF and YF peaked at 105 DABB. The expression of miRNA166 in YF was significantly higher than in CF at 45, 105, and 125 DABB. The expression of miRNA171 in CF and YF increased from 45 to 65, decreased from 85 to 125, and then increased to a high level at 145 DABB. The potential target of miRNA171, *MdHAM*, fluctuated in its expression level from 45 to 105 DABB, and was then downregulated after 125 DABB. The expression of miRNA171 in YF was significantly lower than CF from 65 to 145 DABB.

### RT-qPCR of differentially expressed miRNAs and their targets involved in internode elongation

**Figure 8:**
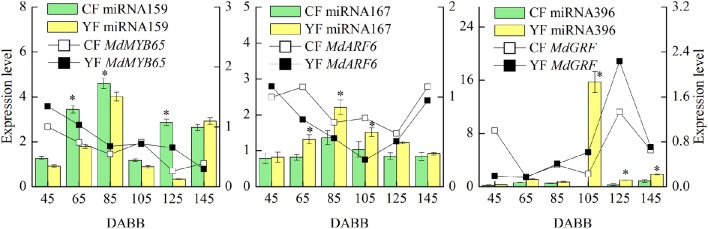
**Differentially expressed miRNAs and their targets involved in internode elongation between CF and YF**. ^*^Indicates *P* < 0.05, Student's *t*-test; Error bars indicate SE.

MiRNAs and their targets, which are putatively involved in internode elongation, that were differentially expressed in YF vs. CF are shown in Figure 8. The expression of miRNA159 in YF was significantly lower at 65, 85, and 125 DABB than it was in CF. The potential target, *MdMYB65*, was downregulated from 45 to 145 DABB. The highest expression level of miRNA167 in both CF and YF was observed during the slow growth period at 80 DABB. The potential target, *MdARF6*, was downregulated from 45 to 125 DABB, and upregulated at 145 DABB. The expression of miRNA167 in YF was significantly higher than in CF at 65, 85, and 105 DABB. The expression of miRNA396 in YF peaked at 105 DABB. The expression of miRNA396 in YF was significantly higher than in CF at 105, 125, and 145 DABB. The miRNA396 potential target gene, *MdGRF*, exhibited a high level during the later growth stage. The expression of miRNA159, miRNA167, and miRNA396 in YF and CF shoot tips exhibited their highest level of expression during the period of slow shoot growth. These data suggest that these miRNAs inhibited cell division in developing shoots.

### Expression of cell cycle- and cell elongation-related genes

**Figure 9:**
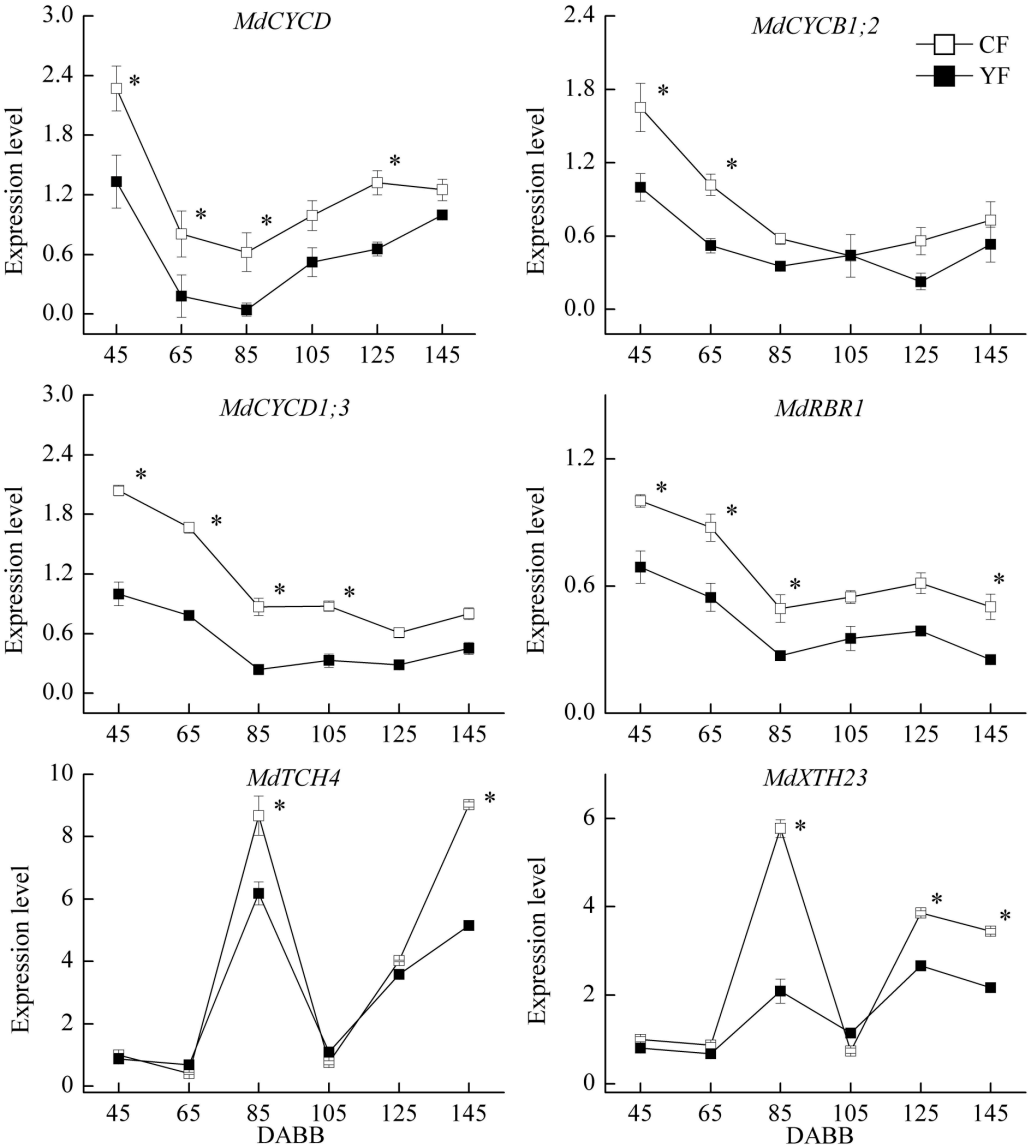
**Expression of cell cycle and cell elongation related genes**. ^*^Indicates *P* < 0.05, Student's *t*-test; Error bars indicate SE.

A strong correlation has been observed between final internode length and cell number (Brown and Sommer, 1992). Therefore, the expression of cell cycle and cell elongation related genes were examined (Figure 9). The expression of the cell cycle related genes, *MdCYCD, MdCYCB1.2, MdCYCD1.3*, and *MdRBR1* exhibited high levels of transcript abundance during the period of rapid growth after bud break, and were downregulated during the period of slow growth, followed by a slight upregulation at the later stages of sampling. *MdCYCD* expression in YF was significantly lower than in CF from 45 to 125 DABB. Expression levels of *MdCYCB1;2, MdCYCD1;3*, and *MdRBR1* genes in YF were significantly lower than in CF during the period of rapid growth just after bud break. The cell elongation genes, *MdTCH4* and *MdXTH23*, exhibited high expression levels during the period of slow growth and in the autumn. *MdTCH2* and *MdXTH23* expression in YF exhibited a lower expression level than in CF during these periods.

### Expression of hormone-related genes

**Figure 10:**
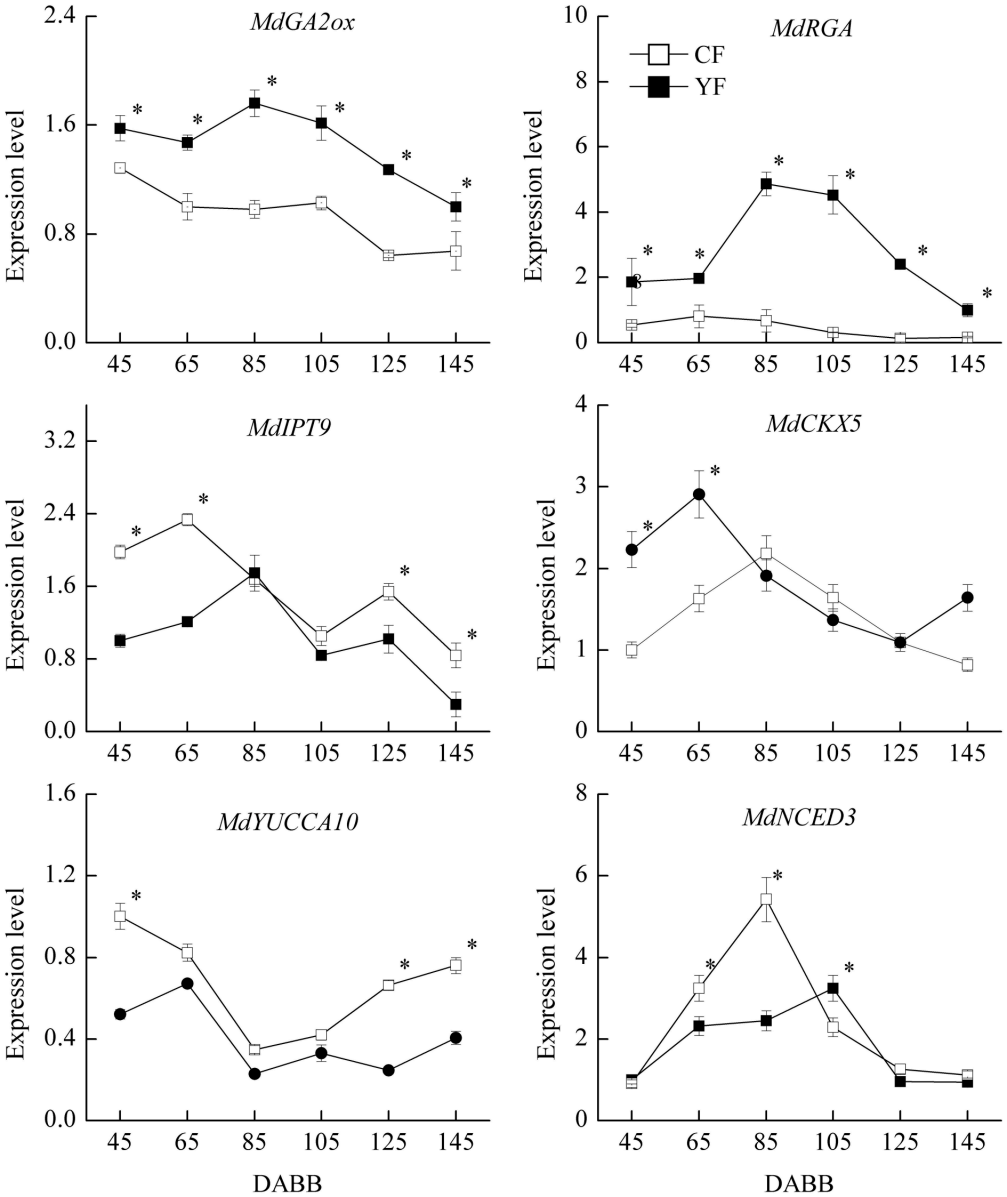
**Expression of hormone related genes**. ^*^Indicates *P* < 0.05, Student's *t*-test; Error bars indicate SE.

Based on the observed effect of plant hormones on growth, the expression of hormone-related genes was also examined (Figure 10). The expression of the *GA2ox* gene, which catalyzes the catabolism of bioactive GA or their precursors, was significantly higher in YF than in CF. In addition, the GA signal transduction gene, *MdRGA*, which negatively regulates GA response, was also more highly expressed in YF than in CF, especially at 80 and 105 DABB. The expression of the cytokinin synthesis gene, *MdIPT9*, was significantly lower in YF than in CF during the period of rapid growth at 45, 65, 125, and 145 DABB. The *MdCKX5* gene, which is involved in the breakdown and catabolism of cytokinin, was upregulated to a higher level in YF than in CF at 45 and 65 DABB. The expression of the ABA synthesis gene, *MdNCED3*, was significantly lower in YF than in CF at 65 and 85 DABB, but higher in YF than in CF at 105 DABB.

### Effect of GA on shoot growth and miRNA expression

**Figure 11:**
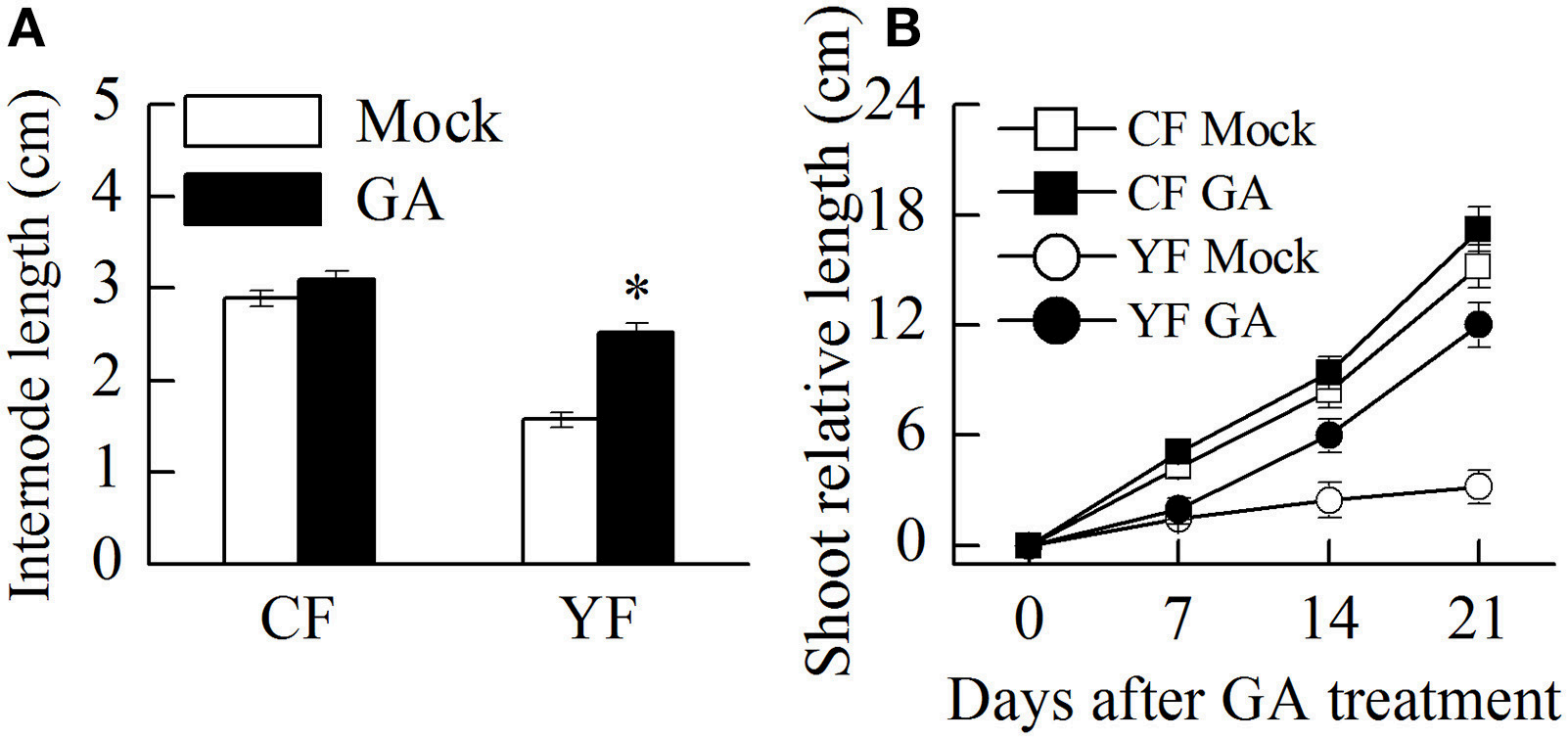
**Effect of GA treatment on shoot and internode growth. (A)** Effect of GA treatment on internode length. **(B)** Effect of GA treatment on shoot growth. ^*^Indicates *P* < 0.05, Student's *t*-test; Error bars indicate SE.

Auxin and GA_4+7_ was applied to YF and CF trees. Results indicated that the application of GA promoted shoot growth and internode elongation in YF trees (Figure 11A). The terminal shoot started to grow rapidly 1 week after the GA treatment was applied (Figure 11B). In total, internode length in YF trees increased by about 33% after the GA treatment, however, this was still less than the internode length in CF trees (Figure 11A). The auxin application did not stimulate shoot growth in either YF or CF trees (data not shown).

**Figure 12:**
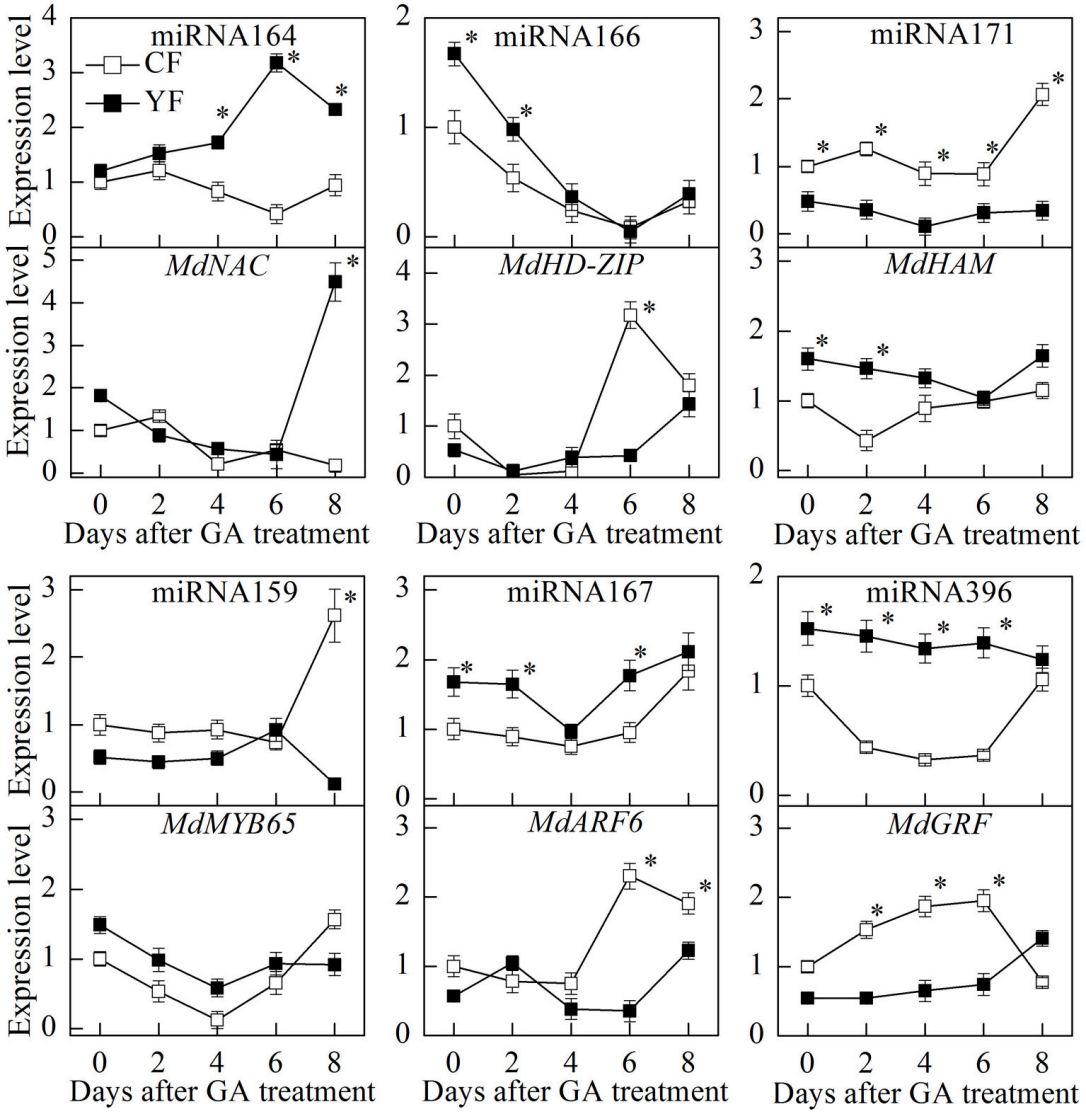
**Expression of key miRNA after GA spraying**. ^*^Indicates *P* < 0.05, Student's *t*-test; Error bars indicate SE.

The GA treatment dramatically downregulated miRNA166 expression (Figure 12). The potential target of miRNA166, *MdHD-ZIP*, was also downregulated during the first 2 days after the GA treatment. After the 6th day, however, *MdHD-ZIP* was upregulated. During the first 4 days after the GA treatment, the expression of miRNA164 in CF and YF trees was slightly upregulated. Then miRNA164 was downregulated in CF, but upregulated in YF. The expression of miRNA171 in YF trees exhibited little response to the GA treatment. The expression of miRNA171 in CF trees was upregulated on the 8th day after the application of the GA treatment. The expression of miRNA159 in both YF and CF trees showed little response to the GA treatment during the first 6 days, however, on the 8th day, the miRNA159 was upregulated in CF trees but downregulated in YF trees. Relative to the expression levels in CF trees, miRNA396 expression in YF trees slowly went down after the application of the GA treatment. The expression of miRNA167 exhibited little response during the first 2 days after the GA application but was downregulated on the 6th day, and then subsequently upregulated.

## Discussion

YF is a natural spur mutant. YF trees have short internodes and reduced shoot length. Whether or not spur-type apple trees are also a fruiting characteristics, has been a source of debate, especially since the spur type phenotype of siblings derived from the spur-type apple exhibiting great variability (Fideghelli et al., 2003). Regardless, spur-type apple cultivars undoubtedly moderately reduced tree size as compared to standard-type trees (Costes et al., 2010). Unlike dwarfing rootstocks that reduce the proportion of lateral buds that develop into long shoots during the early development of trees (Lauri and Trottier, 2006; Seleznyova et al., 2008), YF produces a dwarf tree architecture by reducing the duration of shoot vegetative growth and shortening internode length.

In the present study, YF trees were demonstrated to contain lower levels of GA, ZR, IAA, and ABA. An application of GA promoted cell division in the shoot apical meristem and an increase in internode elongation in YF trees (Figures 11A,B). In contrast, YF trees exhibited no response to IAA. Previous studies have reported that the application of 6-benzylaminopurine and GA to apple nursery trees increased shoot elongation to a much greater extent than applying 6-benzylaminopurine alone (Zhang et al., 2011; Doric et al., 2015). Hence, among the hormones, GA, ZR, IAA, and ABA, it appears that GA plays a major role in YF trees.

The miRNA sequencing conducted in the present study confirmed the presence of 202 known miRNAs expression in apple shoot tips and discovered an additional 498 novel miRNAs. A total of 42 known miRNAs and 93 novel miRNAs were differentially expressed in the shoot tips of CF and YF trees. The differential expression of the miRNAs appeared to be related to the differences in shoot development observed in the two genotypes. This supports the premise that miRNAs are involved in the regulation of shoot architecture in YF trees.

Internode length and the number of nodes are two essential components that determine plant height. Compared to internode length, however, the impact of shoot apical meristem activity has a greater effect on shoot development. For example, the shoot apical meristem is responsible for terminating cell division when trees enter dormancy or when the vegetative meristem is converted to a floral meristem. The number of nodes is also determined by continued cell divisions in the shoot apical meristem. Therefore, factors affecting SAM development would also affect the number of nodes produced on a shoot during an annual growth cycle. Our results indicate that miRNA164, miRNA166, and miRNA171 are potentially involved in SAM development and differentially expressed in YF vs. CF apple shoot tips. A degradation of the NAC-domain transcription factors, CUC1 and CUC2 (Mallory et al., 2004; Raman et al., 2008) by miRNA164 has been reported to constrain the expansion of the boundary domain by degrading the NAC-domain transcription factors, CUC1 and CUC2 (Mallory et al., 2004; Raman et al., 2008). In *Arabidopsis*, CUC1, CUC2, and CUC3 function redundantly in initiating SAM and establishing organ boundaries (Aida et al., 1997). Abolishing miR164 regulation of CUC2 resulted in progressive enlargement of the boundary domain (Raman et al., 2008). The shoot tips in YF trees exhibited abnormal shoot tips, with the dome of the apex appearing a little larger than the apical dome in CF trees. This phenotype is also present in apples with columnar architecture and in some dwarfing rootstocks (Petersen and Krost, 2013). The *mir164abc* mutant frequently displayed extremely short internodes, small floral organs, and reduced fertility. Thus, miRNA164 may be involved in abnormal shoot and internode development in YF trees.

Among all of the miRNAs, miRNA166 had the highest level of expression in the shoot tips. miRNA166 potentially inhibited SAM development by targeting a set of HD-ZIP transcription factors that are important for maintaining pluripotency in shoot apical meristems (Williams et al., 2005). AGO10 binds miRNA165/166 to prevent its association with the broadly expressed AGO1 and thus prevents the degradation of HD-ZIP transcription factors in the SAM (Zhou et al., 2015). YF trees have a high proportion of spur shoots. Many of the lateral shoots in YF trees stopped elongating soon after bud break. Thus, miRNA166 may be involved in apple regulating the spur shoot habit in apple trees.

miRNA171 potentially targets the HAM subfamily genes, *SCL6-II, SCL6-III*, and *SCL6-IV*, within the *GRAS* gene family. miRNA171 affects the maintenance of SAM indeterminacy by regulating the shoot apical meristem WUS-CLV feedback loop (Schulze et al., 2010). The expression level of miRNA171 was significantly lower in YF trees than in CF trees. The height of *Arabidopsis* plants overexpressing miR171c was significantly higher than wild-type plants (Long et al., 2010). Upregulation of OsMIR171c in a delayed heading mutant rice genotype exhibited pleiotropic phenotypic defects, including a prolonged vegetative phase and a delayed heading date (Fan et al., 2015). These observations correspond to the reduced growth in YF shoots, relative to shoot growth in CF trees, and the earlier date in which growth ceased in YF trees, relative to CF trees.

As a result, it appears that the final internode phenotype results from an interplay between cell division and elongation that progresses in an inverse directions. Cell number has a more predominant role in regulating the growth and development of internodes because of cell length increases only two to three times, but cell numbers increase by 10 to 30 times (Pallardy, 2008). Analysis of the expression pattern of cell cycle genes further supports the premise that the SAM in YF trees has a lower rate of cell division than in CF trees (Figure 12). The expression of *MdCYCD, MdCYCB1;2, MdCYCD1;3*, and *MdRBR1* was significantly lower in YF shoot tips than in CF shoot tips. The period of high expression of the cell cycle genes corresponded to the period of rapid shoot growth; suggesting that cell division may play an instrumental role in shoot growth, and more so than cell elongation. Ripetti et al. (2008) also found that genetic variation in internode length could be primarily attributed to cell numbers, while cell length played more of a secondary role. The miRNA sequencing and GO annotation in the present study identified several miRNAs related to cell division.

miRNA159 potentially deregulates its target genes, MYB33 and MYB65, in vegetative tissues, and inhibit growth by reducing cell proliferation (Alonso-Peral et al., 2010). The expression of miRNA159 was significantly higher in CF shoot tips than in YF shoot tips. The miRNA159 loss of function mutant, *mir159ab*, has an enlarged shoot apical meristem and smaller lateral organs (Alonso-Peral et al., 2010). miRNA159 and its targets have also been reported to be involved in GA-mediated flowering (Gocal et al., 2001; Achard et al., 2004). The expression of miRNA159 in YF shoot tips exhibited little response to the application of GA during the first 6 days after spraying GA. Interestingly, miR159a and miR159b also remained unchanged after the application of GA in wild-type *Arabidopsis* (Alonso-Peral et al., 2010). The potential target gene of miR159a, MYB65, however, was downregulated after the application of GA in *Malus domestica*. Thus, GA maybe regulate the expression of the target of miRNA159 to produce the miRNA159 phenotype.

miRNA396 negatively regulates its potential target gene, GROWTH RESPONDING FACTOR (GRF), to affect cell division in shoot meristems, leaves, and roots. miRNA396 appears to act as a strict repressor of the GA and CK pathways, and negatively regulates cell proliferation by decreasing cell division activity and the expression of cell cycle-related genes (Wang et al., 2010). The potential downregulation of the GA pathway genes by miR396 corresponds to the dwarf phenotype observed in miR396 overexpressing lines (Liu et al., 2009). The high level of expression of miRNA396 in YF shoot tips may inhibit cell division and internode elongation. miRNA396 was downregulated after GA application. Thus, GA may affect downstream GA response by downregulating miRNA396.

miRNA167 regulates vegetative and reproductive growth by controlling the expression of auxin response factors 6 and 8 (ARF6/8) in plants. Overexpression of miRNA167a results in twisted leaves, short inflorescences, and arrested flower development; thereby fully producing the mature phenotype of arf6arf8 plants (Wu et al., 2006). The expression of miRNA167 was significantly higher in YF shoot tips than in CF shoot tips. GA treatment downregulated miRNA167 at first, and then subsequently induced it; resulting in upregulating. But GA also increased the expression of the target gene, ARF6. DELLA has been reported to interact with ARF6 and inhibit ARF6 DNA-binding to modulate ARF6 gene expression (Oh et al., 2013). Hence, GA may promote cell elongation mechanisms in YF trees that involve miRNA167-ARF-mediated responses.

**Figure 13:**
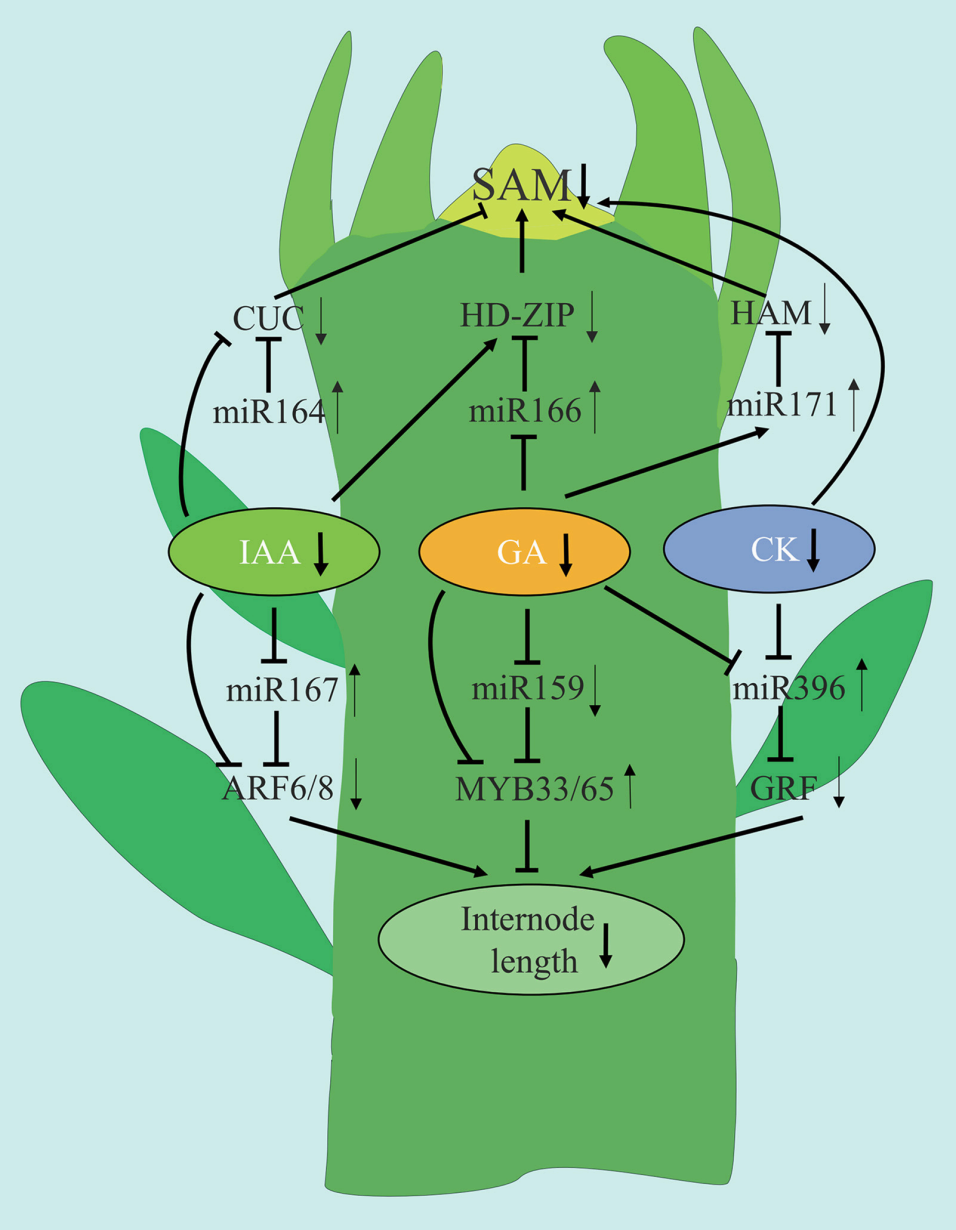
**Tentative model for the association of miRNAs with the phytohormone crosstalk regulation the spur type apple tree architecture**. Diagram represents a schematic drawing of an apple shoot apex. Arrows and inhibition lines linking the gene names represent positive and negative interactions, respectively. Up and down arrows behind the gene names represent up and down regulation, respectively.

Based upon the findings of the current study, we present a model to explain how plant hormones and miRNA are involved in regulating the shoot development in spur-type YF trees (Figure 13). Low levels of IAA, GA, and CK are associated with reduced shoot growth in YF trees. GA appears to play a major role in regulating shoot development. Low levels of GA in YF shoots may reduce the number of cell divisions in the SAM, relative to the SAM in CF shoots, thus causing shoot growth to cease much earlier in the YF trees as compared to in the CF trees. In the model, low expression of miRNA164, miRNA171, and high expression of miRNA166 and their potential target genes affected SAM development and reduced the duration of shoot growth in YF trees. Of the three miRNAs, the regulation of miRNA166 by GA appeared to play a major role in shoot tips elongation in YF trees. The high levels of expression of miRNA167 and miRNA396, and the low expression of miRNA159, in YF trees appears to have inhibited cell division and reduced internode length. The results of the present study, and the hypothetical model presented, contributes to a better understanding of shoot development in perennial plants; especially those that exhibit a spur-type growth architecture.

## Author contributions

CS, DZ, JM, and MH participate in the experimental design. CS, JZ, LZ, and WL participate in material sampling, field measurements and the laboratory data measurement. CS, BZ, YL, and GL participate in the laboratory data measurement. CS, DZ, JM, and MH participate in the paper writing and discussion. All authors reviewed the manuscript.

### Conflict of interest statement

The authors declare that the research was conducted in the absence of any commercial or financial relationships that could be construed as a potential conflict of interest.

## Supplementary material

Figure S1 **The meteorological condition of the experiment site**.

Figure S2 **Size distribution of novel miRNAs and the identity of the first nucleotide**.

Table S1 **Quality evaluation of CF and YF sequencing data**.

Table S2 **Distribution of small RNAs among different categories in CF and YF libraries**.

Table S3 **Character of known miRNA**.

Table S4 **Character of novel miRNA**.

Table S5 **Prediction target gene of known miRNA**.

Table S6 **Prediction target gene of novel miRNA**.

Table S7 **Distribution of different RNAs categories in degradome library**.

Table S8 **GO functional analysis of identified targets of known miRNAs**.

Table S9 **Known miRNA KEGG pathway analysis**.

Table S10 **GO functional analysis of identified targets of novel miRNAs**.

Table S11 **Novel miRNA KEGG pathway analysis**.

Table S12 **Primer of microRNA reverse transcription and real time Q-PCR**.

Table S13 **Primer of mRNA for real time Q-PCR**.

## References

[B1] Achard P. Herr A. Baulcombe D. C. Harberd N. P. (2004). Modulation of floral development by a gibberellin-regulated microRNA. Development 131, 3357–3365. 10.1242/dev.01206 15226253

[B2] Aida M. Ishida T. Fukaki H. Fujisawa H. Tasaka M. (1997). Genes involved in organ separation in *Arabidopsis*: an analysis of the cup-shaped cotyledon mutant. Plant Cell 9, 841–857. 10.1105/tpc.9.6.841 9212461 PMC156962

[B3] Alonso-Peral M. M. Li J. Li Y. Allen R. S. Schnippenkoetter W. Ohms S.. (2010). The MicroRNA159-Regulated GAMYB-like genes inhibit growth and promote programmed cell death in *Arabidopsis*. Plant Physiol. 154, 757–771. 10.1104/pp.110.160630 20699403 PMC2949021

[B4] Bonnet E. Wuyts J. Rouzé P. Van de Peer Y. (2004). Evidence that microRNA precursors, unlike other non-coding RNAs, have lower folding free energies than random sequences. Bioinformatics 20, 2911–2917. 10.1093/bioinformatics/bth374 15217813

[B5] Brown C. L. Sommer H. E. (1992). Shoot growth and histogenesis of trees possessing diverse patterns of shoot development. Am. J. Bot. 79, 335–346. 10.2307/2445024

[B6] Chang S. J. Puryear J. Cairney J. (1993). A simple and efficient method for isolating RNA from pine trees. Plant Mol. Biol. Rep. 11, 113–116. 10.1007/BF02670468

[B7] Chen C. Ridzon D. A. Broomer A. J. Zhou Z. Lee D. H. Nguyen J. T.. (2005). Real-time quantification of microRNAs by stem-loop RT-PCR. Nucleic Acids Res. 33, e179. 10.1093/nar/gni178 16314309 PMC1292995

[B8] Chen X. (2005). microRNA biogenesis and function in plants. FEBS Lett. 579, 5923–5931. 10.1016/j.febslet.2005.07.071 16144699 PMC5127707

[B9] Costes E. Lauri P. É. Regnard J. L. (2010). Analyzing fruit tree architecture: implications for tree management and fruit production. Hortic. Rev. 32, 1–61. 10.1002/9780470767986.ch1

[B10] Debat H. J. Ducasse D. A. (2014). Plant microRNAs: recent advances and future challenges. Plant Mol. Biol. Rep. 32, 1257–1269. 10.1007/s11105-014-0727-z

[B11] Dobrev P. I. Havlíček L. Vágner M. Malbeck J. Kamínek M. (2005). Purification and determination of plant hormones auxin and abscisic acid using solid phase extraction and two-dimensional high performance liquid chromatography. J. Chromatogr.A. 1075, 159–166. 10.1016/j.chroma.2005.02.091 15974129

[B12] Doric M. Magazin N. Milic B. Keserovic Z. (2015). Enhancing feathering of one-year-old Gala and Jonagold apple trees through application of 6-benzylaminopurine and gibberellins. Bulgarian J. Agric. Sci. 21, 631–637.

[B13] Fan T. Li X. Yang W. Xia K. Ouyang J. Zhang M. (2015). Rice osa-miR171c mediates phase change from vegetative to reproductive development and shoot apical meristem maintenance by repressing four OsHAM transcription factors. PLoS ONE 10:e0125833. 10.1371/journal.pone.0125833 26023934 PMC4449180

[B14] Fideghelli C. Sartori A. Grassi F. (2003). Fruit Tree Size and Architecture, 622nd Edn. Leuven: International Society for Horticultural Science (ISHS).

[B15] Gallavotti A. (2013). The role of auxin in shaping shoot architecture. J. Exp. Bot. 64, 2593–2608. 10.1093/jxb/ert141 23709672

[B16] Gao C. Ju Z. Cao D. Zhai B. Qin G. Zhu H.. (2015). MicroRNA profiling analysis throughout tomato fruit development and ripening reveals potential regulatory role of RIN on microRNAs accumulation. Plant Biotechnol. J. 13, 370–382. 10.1111/pbi.12297 25516062

[B17] Gocal G. F. Sheldon C. C. Gubler F. Moritz T. Bagnall D. J. Macmillan C. P.. (2001). GAMYB-like genes, flowering, and gibberellin signaling in *Arabidopsis*. Plant Physiol. 127, 1682–1693. 10.1104/pp.010442 11743113 PMC133573

[B18] Guo S. Xu Y. Liu H. Mao Z. Zhang C. Ma Y.. (2013). The interaction between OsMADS57 and OsTB1 modulates rice tillering via DWARF14. Nat. Commun. 4, 67–88. 10.1038/ncomms2542 23463009 PMC3615354

[B19] Dobrev P. I. Kamınek M. (2002). Fast and efficient separation of cytokinins from auxin and abscisic acid and their purification using mixed-mode solid-phase extraction. J Chromatogr. A. 950, 21–29. 10.1016/S0021-9673(02)00024-9 11990994

[B20] Jain M. Chevala V. V. S. N. Garg R. (2014). Genome-wide discovery and differential regulation of conserved and novel microRNAs in chickpea via deep sequencing. J. Exp. Bot. 65, 5945–5958. 10.1093/jxb/eru333 25151616 PMC4203128

[B21] Jiao Y. Wang Y. Xue D. Wang J. Yan M. Liu G.. (2010). Regulation of OsSPL14 by OsmiR156 defines ideal plant architecture in rice. Nat. Genet. 42, 541–544. 10.1038/ng.591 20495565

[B22] Jung S. Ficklin S. P. Lee T. Cheng C. H. Blenda A. Zheng P.. (2014). The Genome Database for Rosaceae (GDR): year 10 update. Nucleic Acids Res. 42, 1237–1244. 10.1093/nar/gkt1012 24225320 PMC3965003

[B23] Kyozuka J. (2007). Control of shoot and root meristem function by cytokinin. Curr. Opin. Plant Biol. 10, 442–446. 10.1016/j.pbi.2007.08.010 17904411

[B24] Lapins K. O. Lapins K. O. (1969). Segregation of compact growth types in certain apple seedling progenies. Can. J. Plantence 49, 765–768. 10.4141/cjps69-130

[B25] Laufs P. Peaucelle A. Morin H. Traas J. (2004). MicroRNA regulation of the CUC genes is required for boundary size control in Arabidopsis meristems. Development 131, 4311–4322. 10.1242/dev.01320 15294871

[B26] Lauri P. R. Trottier C. (2006). Architecture and size relations: an essay on the apple (*Malus* x *domestica*, Rosaceae) tree. Am. J. Bot. 93, 357–368. 10.3732/ajb.93.3.357 21646196

[B27] Leibfried A. To J. Busch W. Stehling S. Kehle A. Demar M.. (2006). WUSCHEL controls meristem function by direct regulation of cytokinin-inducible response regulators. Nature 438, 1172–1175. 10.1038/nature04270 16372013

[B28] Li D. Wang L. Liu X. Cui D. Chen T. Zhang H.. (2013). Deep sequencing of maize small RNAs reveals a diverse set of MicroRNA in dry and imbibed seeds. PLoS ONE 8:e55107. 10.1371/journal.pone.0055107 23359822 PMC3554676

[B29] Liu D. Song Y. Chen Z. Yu D. (2009). Ectopic expression of miR396 suppresses GRF target gene expression and alters leaf growth in *Arabidopsis*. Physiol. Plant. 136, 223–236. 10.1111/j.1399-3054.2009.01229.x 19453503

[B30] Livak K. J. Schmittgen T. D. (2001). Analysis of relative gene expression data using real-time quantitative PCR and the 2^−ΔΔ^^C^_T_ method. Methods 25, 402–408. 10.1006/meth.2001.1262 11846609

[B31] Long W. Mai Y. X. Zhang Y. C. Qian L. Yang H. Q. (2010). MicroRNA171c-targeted SCL6-II, SCL6-III, and SCL6-IV genes regulate shoot branching in *Arabidopsis*. Mol. Plant 3, 794–806. 10.1093/mp/ssq042 20720155

[B32] Mallory A. C. Dugas D. V. Bartel D. P. Bartel B. (2004). MicroRNA regulation of NAC-domain targets is required for proper formation and separation of adjacent embryonic, vegetative, and floral organs. Curr. Biol. 14, 1035–1046. 10.1016/j.cub.2004.06.022 15202996

[B33] Mishra A. K. Agarwal S. Jain C. K. Rani V. (2009). High GC content critical parameter for predicting stress regulated miRNAs in *Arabidopsis thaliana*. Bioinformation 4, 151–154. 10.6026/97320630004151 20198191 PMC2825596

[B34] Niu Q. Li J. Cai D. Qian M. Jia H. Bai S.. (2015). Dormancy-associated MADS-box genes and microRNAs jointly control dormancy transition in pear (*Pyrus pyrifolia* white pear group) flower bud. J. Exp. Bot. 67, 239–257. 10.1093/jxb/erv454 26466664 PMC4682432

[B35] Oh E. Zhu J. Y. Bai M. Y. Arenhart R. A. Sun Y. Wang Z. Y. (2013). Cell elongation is regulated through a central circuit of interacting transcription factors in the *Arabidopsis* hypocotyl. Elife 3:e03031. 10.7554/eLife.03031 24867218 PMC4075450

[B36] Pallardy G. S. (2008). Physiology of Woody Plants. San Diego, CA: Elsevier Inc.

[B37] Petersen R. Krost C. (2013). Tracing a key player in the regulation of plant architecture: the columnar growth habit of apple trees (*Malus* × *domestica*). Planta 238, 1–22. 10.1007/s00425-013-1898-9 23695821

[B38] Raman S. T. G. Peaucelle A. Blein T. Laufs P. Theres K. (2008). Interplay of miR164, CUP-SHAPED COTYLEDON genes and LATERAL SUPPRESSOR controls axillary meristem formation in *Arabidopsis thaliana*. Plant J. 55, 65–76. 10.1111/j.1365-313X.2008.03483.x 18346190

[B39] Ripetti V. Escoute J. Verdeil J. L. Costes E. (2008). Shaping the shoot: the relative contribution of cell number and cell shape to variations in internode length between parent and hybrid apple trees. J. Exp. Bot. 59, 1399–1407. 10.1093/jxb/ern049 18390886

[B40] Schulze S. Schäfer B. N. Parizotto E. A. Voinnet O. Theres K. (2010). *LOST MERISTEMS* genes regulate cell differentiation of central zone descendants in *Arabidopsis* shoot meristems. Plant J. 64, 668–678. 10.1111/j.1365-313X.2010.04359.x 21070418

[B41] Seleznyova A. N. Tustin D. S. Thorp T. G. (2008). Apple dwarfing rootstocks and interstocks affect the type of growth units produced during the annual growth cycle: precocious transition to flowering affects the composition and vigour of annual shoots. Ann. Bot. 101, 679–687. 10.1093/aob/mcn007 18263898 PMC2710180

[B42] Song Y. Zhang Y. M. Liu J. Wang C. Z. Liu M. Y. Feng S. Q. (2012). Comparison of gibberellin acid content and the genes relatived to GA biosynthesis between ‘Changfu 2’ apple (*Malus domestica* Borkh.) and its spur sport. Sci. Agric. Sin. 45, 2668–2675.

[B43] Steffens G. L. Hedden P. (1992). Comparison of growth and gibberellin concentrations in shoots from orchard-grown standard and thermosensitive dwarf apple trees. Physiol. Plant. 86, 544–550. 10.1111/j.1399-3054.1992.tb02168.x

[B44] Taylor R. S. Tarver J. E. Hiscock S. J. Donoghue P. C. J. (2014). Evolutionary history of plant microRNAs. Trends Plant Sci. 19, 175–182. 10.1016/j.tplants.2013.11.008 24405820

[B45] Wang L. Gu X. Xu D. Wang W. Wang H. Zeng M.. (2010). miR396-targeted AtGRF transcription factors are required for coordination of cell division and differentiation during leaf development in *Arabidopsis*. J. Exp. Bot. 62, 761–773. 10.1093/jxb/erq307 21036927 PMC3003814

[B46] Williams L. Grigg S. P. Xie M. Christensen S. Fletcher J. C. (2005). Regulation of *Arabidopsis* shoot apical meristem and lateral organ formation by microRNA miR166g and its AtHD-ZIP target genes. Development 132, 3657–3668. 10.1242/dev.01942 16033795

[B47] Wu J. Wang D. F. Liu Y. F. Wang L. Qiao X. Zhang S. L. (2014). Identification of miRNAs involved in pear fruit development and quality. BMC Genomics 15:953. 10.1186/1471-2164-15-953 25366381 PMC4233070

[B48] Wu M. F. Tian Q. Reed J. W. (2006). *Arabidopsis microRNA167* controls patterns of *ARF6* and *ARF8* expression, and regulates both female and male reproduction. Development 133, 4211–4218. 10.1242/dev.02602 17021043

[B49] Xia R. Zhu H. An Y. Q. Beers E. P. Liu Z. (2012). Apple miRNAs and tasiRNAs with novel regulatory networks. Genome Biol. 13, 1–18. 10.1186/gb-2012-13-6-r47 22704043 PMC3446319

[B50] Yang P. F. Hao Y. Y. Tian C. F. (2000). Studies on the anatomy the vessel member of spur-type apple. Acta Hortic. Sin. 27, 52–54.

[B51] You C. X. Qiang Z. Wang X. F. Xie X. B. Feng X. M. Zhao L. L.. (2014). A dsRNA-binding protein MdDRB1 associated with miRNA biogenesis modifies adventitious rooting and tree architecture in apple. Plant Biotechnol. J. 12, 183–192. 10.1111/pbi.12125 24119151

[B52] Zhang B. H. Pan X. P. Cannon C. H. Cobb G. P. Anderson T. A. (2006). Conservation and divergence of plant microRNA genes. Plant J. 46, 243–259. 10.1111/j.1365-313X.2006.02697.x 16623887

[B53] Zhang Q. W. Song C. H. Xing L. B. Han M. Y. Zhao C. P. Gao-Chao L. I. (2011). Effects of 6-BA and GA_(4+7) as well as other manual measures on branches formation of apple nursery stock. J. Fruit Sci. 28, 1071–1076.

[B54] Zhang Y. C. Yu Y. Wang C. Y. Li Z. Y. Liu Q. Xu J.. (2013). Overexpression of microRNA OsmiR397 improves rice yield by increasing grain size and promoting panicle branching. Nat. Biotechnol. 31, 848–852. 10.1038/nbt.2646 23873084

[B55] Zhou Y. Honda M. Zhu H. Zhang Z. Guo X. Li T. (2015). Spatiotemporal sequestration of miR165/166 by *Arabidopsis* argonaute10 promotes shoot apical meristem maintenance. Cell Rep. 341, 1819–1827. 10.1016/j.celrep.2015.02.047 25801022

[B56] Zhu H. Xia R. Zhao B. An Y. Dardick C. D. Callahan A. M.. (2012). Unique expression, processing regulation, and regulatory network of peach (prunus persica) miRNAs. BMC Plant Biol. 12:149. 10.1186/1471-2229-12-149 22909020 PMC3542160

