# Supplementary figures and images for: miRNA and Degradome Sequencing Reveal miRNA and Their Target Genes That May Mediate Shoot Growth in Spur Type Mutant “Yanfu 6”
Source: Front Plant Sci. 2017 Mar 30;8:441. doi: 10.3389/fpls.2017.00441 (PMC5371658; doi:10.3389/fpls.2017.00441)

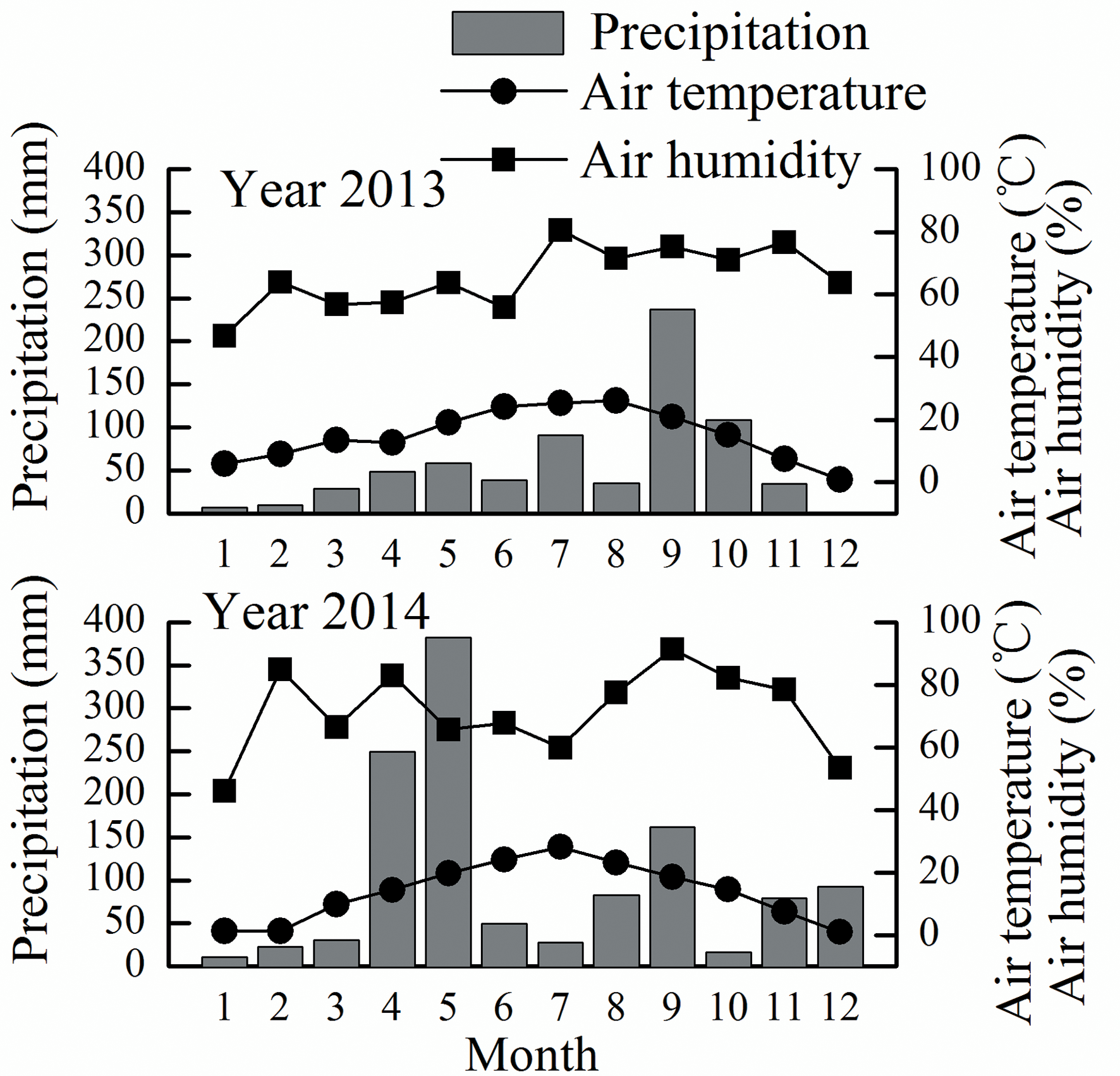

Supplement: Figure S1 — The meteorological condition of the experiment site. [file Image1.TIF]

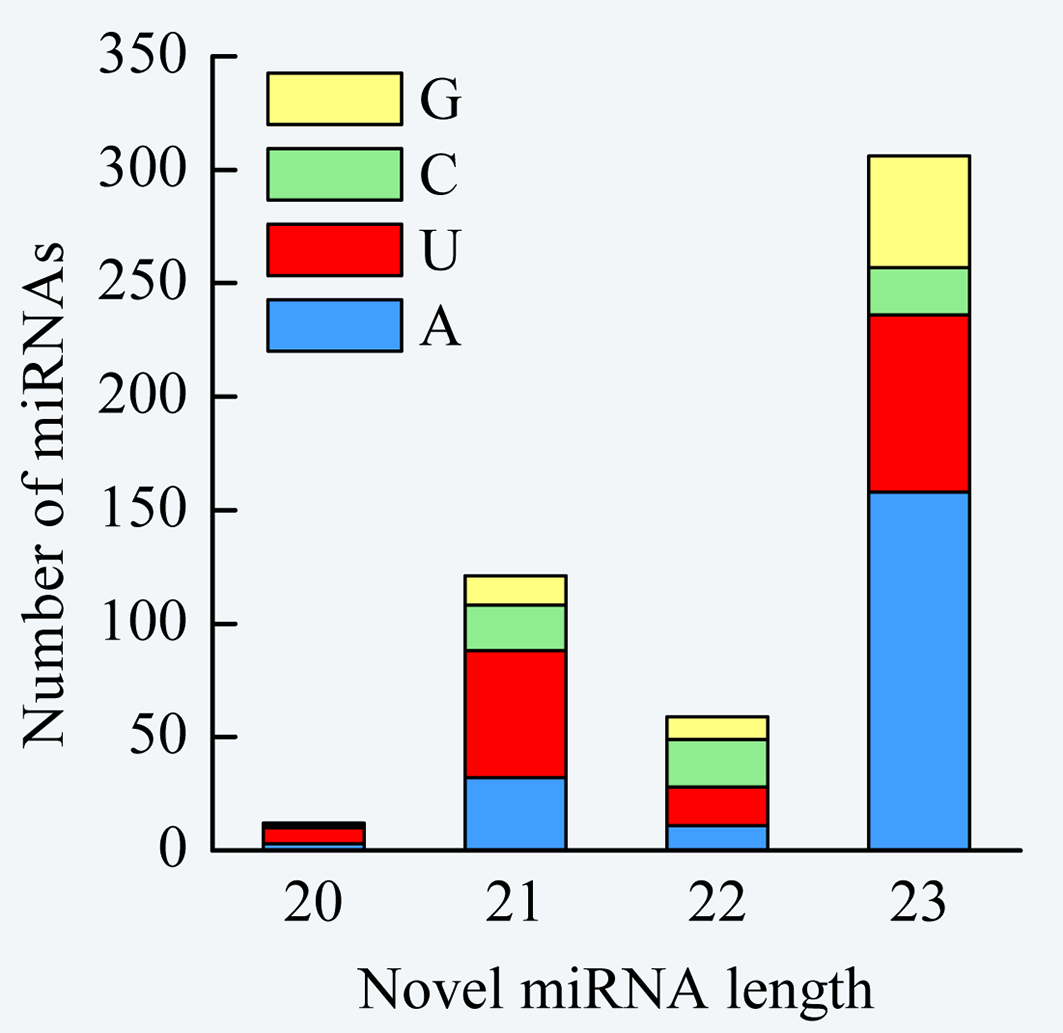

Supplement: Figure S2 — Size distribution of novel miRNAs and the identity of the first nucleotide. [file Image2.TIF]
